# Direct biological fixation provides a freshwater sink for N_2_O

**DOI:** 10.1038/s41467-023-42481-2

**Published:** 2023-10-25

**Authors:** Yueyue Si, Yizhu Zhu, Ian Sanders, Dorothee B. Kinkel, Kevin J. Purdy, Mark Trimmer

**Affiliations:** 1 School of Biological and Behavioural Sciences, Queen Mary, University of London, London, E1 4NS UK; 2 School of Life Sciences, University of Warwick, Coventry, CV4 7AL UK

**Keywords:** Element cycles, Environmental sciences

## Abstract

Nitrous oxide (N_2_O) is a potent climate gas, with its strong warming potential and ozone-depleting properties both focusing research on N_2_O sources. Although a sink for N_2_O through biological fixation has been observed in the Pacific, the regulation of N_2_O-fixation compared to canonical N_2_-fixation is unknown. Here we show that both N_2_O and N_2_ can be fixed by freshwater communities but with distinct seasonalities and temperature dependencies. N_2_O fixation appears less sensitive to temperature than N_2_ fixation, driving a strong sink for N_2_O in colder months. Moreover, by quantifying both N_2_O and N_2_ fixation we show that, rather than N_2_O being first reduced to N_2_ through denitrification, N_2_O fixation is direct and could explain the widely reported N_2_O sinks in natural waters. Analysis of the nitrogenase (*nifH*) community suggests that while only a subset is potentially capable of fixing N_2_O they maintain a strong, freshwater sink for N_2_O that could be eroded by warming.

## Abstract (web summary)

Denitrification is still widely considered as the only natural sink for N_2_O here we show how direct biological fixation represents an alternative sink for this potent climate gas.

## Introduction

Nitrous oxide (N_2_O) is a potent climate gas, with ~273 times the global warming potential of carbon dioxide (CO_2_)^1^ and strong ozone-depleting properties^2^. The atmospheric concentration of N_2_O continues to rise through the use of nitrogen-based fertilisers, fossil fuel combustion, biomass burning and sewage discharge^3^ and has already increased by approximately 20% since 1750^4^. Not surprisingly, given its atmospheric potency, research to date has focused on these N_2_O sources with N_2_O sinks being relatively understudied^5–7^. The few studies reporting on both N_2_O sources and sinks^8–11^ often simply document the sinks as concentrations below that expected for water (marine or freshwater) at equilibrium with the atmosphere and the true mechanism behind this N_2_O deficit remains largely unknown.

In terrestrial and aquatic environments, N_2_O can be produced from both microbial nitrification^12^ either via hydroxylamine oxidation (NH_4_^+^ → NH_2_OH → N_2_O), or hybrid formation (NO_2_^−^ + NH_2_OH → N_2_O)^13^, and incomplete denitrification (NO_3_^−^ → NO_2_^−^ → NO→ N_2_O[$\to$ N_2_])^14^. Where oxygen is limiting and/or completely absent, N_2_O can be further reduced to N_2_ in the last step of microbial denitrification (N_2_O→N_2_) that is typically mediated by facultative anaerobic bacteria^14,15^. As such, any undersaturation – indicating a sink for N_2_O – as observed in some waters has routinely been attributed to that last step in denitrification.

However, such N_2_O undersaturation has typically been reported in well-oxygenated, shallow freshwaters^8–10,16–21^ (down to 13% of air equilibration, typically ~70–100%) and surface-ocean-waters^5,11,22–28^ (down to 34%, typically ~90%) where canonical denitrification is unlikely to explain any undersaturation in N_2_O. While N_2_O consumption by denitrification has been reported in both anoxic and oxic-to-anoxic transitioning waters in the Eastern Tropical North Pacific^6^, the reasons for N_2_O undersaturation in general remain poorly understood, with many instances of N_2_O undersaturation remaining unaccounted for^8–10,19–21^ or simply being dismissed as analytical artifacts^24,29^. Further, as N_2_O sources generally increase at higher concentrations of ammonium and nitrate (i.e., fixed, bio-available N)^8,25^, any potential undersaturation in N_2_O could be masked by stronger production of N_2_O from nitrification and denitrification. This might explain why many accounts of N_2_O undersaturation have been reported in N limited environments^5,9,19,21,22^.

In recent years, evidence has been presented for an additional pathway to denitrification for N_2_O reduction, namely – N_2_O dependent N fixation – that has been reported for pure cultures of marine *Trichodesmium* and *Crocosphaera*^5^. N_2_O fixation has also been reported in the surface waters of the Eastern Tropical South Pacific^5,23^, where the measured N_2_O fixation activity could contribute some (0.2 – 60%) of the total N_2_O reduction^5^. As long ago as 1954, it was shown^30^ that ^15^N_2_O could be assimilated by soybean root nodules with activity comparable to ^15^N_2_ assimilation. These findings show that N_2_O fixation (e.g. N_2_O→NH_4_^+^) represents an alternative N_2_O reduction pathway to the terminal step in denitrification (N_2_O→N_2_) that may explain some of the undersaturation reported for N_2_O. Within the widespread accounts of N_2_O undersaturation found in well-oxygenated waters, only a few studies mentioned the possibility of N_2_O fixation^5,22,23^ and it is not widely acknowledged.

Primary production and N_2_ fixation are tightly coupled in N-limited ecosystems^31^. Some early studies (1952–1986) showed that N_2_O is a competitive inhibitor for N_2_ fixation and it could also be a substrate for the enzymatic nitrogenase complex^32–35^, indicating that N_2_O fixation (e.g. N_2_O→NH_4_^+^) may be related to N_2_ fixation (N_2_→NH_4_^+^). Further, with its N≡N bond N_2_ fixation has a high activation energy (~1 to 2 eV vs. 0.65 eV and 0.32 eV for respiration and photosynthesis, respectively)^36,37^ which makes fixing N_2_ in the cold energetically unfavourable. As a consequence, the abundance of diazotrophs has been shown to decrease as temperatures decline^36^. In contrast, the energy required to fix N_2_O (Eq. 1, Δ*G* defined for freshwater at 10 °C, *see* Supplementary Text 1) is lower than that for N_2_ (Eq. 2) and being able to fix N_2_O could confer an ecological advantage to some microbes either in the cold or when resources (light or reduced substrates) in general are limiting.

1 $$\begin{array}{ccc}{0.5{{{{{\rm{N}}}}}}}_{2}{{{{{\rm{O}}}}}}+{1.5{{{{{\rm{H}}}}}}}_{2}{{{{{\rm{O}}}}}}\to {1{{{{{\rm{NH}}}}}}}_{3}+{1{{{{{\rm{O}}}}}}}_{2} & \Delta G=+ 247{{{{{\rm{kJ}}}}}} & ({{{{{\rm{per}}}}}}\,{{{{{{\rm{NH}}}}}}}_{3})\end{array}$$

2 $$\begin{array}{ccc}0.5{{{{{{\rm{N}}}}}}}_{2}+1.5{{{{{{\rm{H}}}}}}}_{2}{{{{{\rm{O}}}}}}\to 1{{{{{{\rm{NH}}}}}}}_{3}+0.75{{{{{{\rm{O}}}}}}}_{2} & \Delta G=+ 291{{{{{\rm{kJ}}}}}} & ({{{{{\rm{per}}}}}}\,{{{{{{\rm{NH}}}}}}}_{3})\end{array}$$

While the ~18% energy saving for fixing N_2_O versus N_2_ is seemingly modest, it is comparable to the recognised 21% saving delivered by assimilating NO_3_^−^ (Eq. 3) rather than fixing N_2_ (ref. ^38^) (see Supplementary Text 1).

3 $$\begin{array}{ccc}{{{{{{\rm{NO}}}}}}}_{3}^{-}+3{{{{{{\rm{H}}}}}}}^{+}+2{{{{{{\rm{e}}}}}}}^{-}\to 1{{{{{{\rm{NH}}}}}}}_{3}+1.5{{{{{{\rm{O}}}}}}}_{2} & \Delta G=+ 241{{{{{\rm{kJ}}}}}} & ({{{{{\rm{per}}}}}}\,{{{{{{\rm{NH}}}}}}}_{3})\end{array}$$

With both the last step in denitrification (N_2_O→N_2_) and N_2_O fixation (N_2_O→NH_4_^+^) providing sinks for N_2_O it is ecologically important to distinguish between these two parts of total N_2_O reduction. Further, any genuine direct N_2_O fixation (N_2_O→NH_4_^+^) needs to be distinguished from indirect N_2_O fixation i.e., that which could occur after the initial reduction of N_2_O to N_2_ (N_2_O→N_2_→NH_4_^+^). Despite the few studies^5,23,30^ documenting N_2_O fixation so far, to the best of our knowledge, there has been no characterisation of N_2_O fixation in relation to canonical N_2_ fixation through the dual use of ^15^N_2_O and ^15^N_2_ in natural communities.

In 2005, we set up 20 experimental ponds (each with 1 m^3^ water volume, 0.5 m depth) in East Stoke, Dorset, UK, to experimentally study the whole-ecosystem effects of climate warming^39–41^. Here, however, we exploited the fact that our experimental ponds are also N-limited^41^, being fed only by rain water, to characterise any potential N_2_O fixation in a controlled, experimental system. Despite being artificial, the ponds have well-established freshwater ecosystems^39–42^ with diverse cyanobacteria communities^42^, among which some Nostocales^43^ and Oscillatoriales^44^ are known to fix N_2_.

Here, we show that the ponds are undersaturated in both N_2_ and N_2_O and further hypothesise that the pond communities fix both gases to support primary production. Then, due to the different energy demands of N_2_ and N_2_O fixation, we hypothesise that the two processes will respond differently to temperature. We use incubations with pond biomass and ^15^N_2_ and ^15^N_2_O stable isotope techniques to quantify their fixation activity, distinguish direct from indirect N_2_O fixation and characterise the temperature dependence of each N-fixing process. Finally, with no known freshwater candidates for N_2_O fixation to date, we explore the recognised N_2_ fixing community in relation to N_2_O fixation. We ask whether: 1, is N_2_O fixation mediated by the total nitrogenase (*nifH*) community simply in relation to the relative availability of N_2_O to N_2_; or 2, is N_2_O fixation preferentially mediated by a subset of the *nifH* community?

## Results

### Contrasting seasonalities in undersaturation for N_2_ and N_2_O

**Fig. 1:**
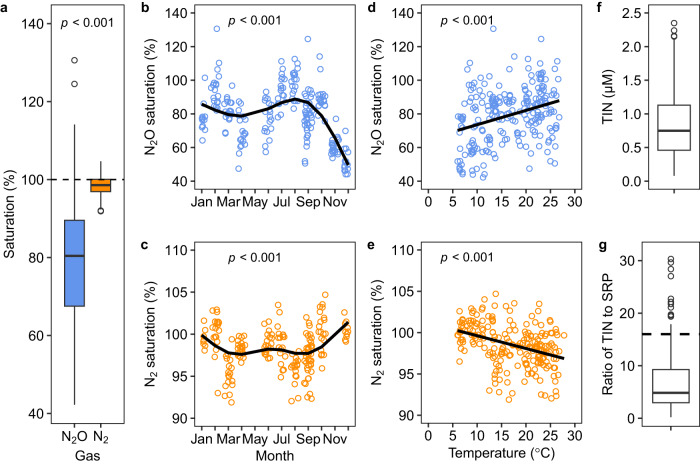
Seasonal and overall levels of N_2_O and N_2_ saturation in the ponds. **a** Box-whisker plots showing overall that the saturation of N_2_O was lower than that for N_2_ in the ponds (*p* < 0.001, two-sided, Supplementary Table 1). The dashed line denotes 100% atmospheric equilibrium for the gases. **b**, **c** show the saturation in N_2_O had a different seasonal pattern compared to N_2_ (Note the different scales on the y-axes). The solid lines in **b** and **c** represent the best fitting GAMM models (two-sided, Supplementary Table 1). **d** N_2_O saturation increased at higher temperatures while, in contrast, **e** N_2_ saturation declined. The lines in **d** and **e** are simple first-order linear regressions (two-sided). **f** Overall concentration of total inorganic nitrogen (TIN) in the ponds (*n* = 213 samples for 11 months in 20 ponds). **g** The ratio of TIN to soluble reactive phosphorous (SRP) in the ponds (*n* = 168 samples for 11 months for 20 ponds, SRP data omitted below the detection limit). The dashed line in **g** denotes the Redfield ratio of N to P of 16:1. Each box in **a**, **f** and **g** shows the 25th to 75th percentiles, horizontal lines the median, open circles denote outliers and whiskers extend to 1.5 times the interquartile range. *n* = 230 and *n* = 215 samples for N_2_O and N_2_ saturation, respectively, for 20 ponds, in 11 months from November 2019 to April 2022 (see Supplementary Table 2).

Concentrations of dissolved N_2_O and N_2_ were both significantly below atmospheric equilibration (*p* < 0.001, Fig. 1a) and the ponds are sinks for both atmospheric N_2_O and N_2_. Overall, N_2_O was more under-saturated than N_2_ (*p* < 0.001, *t* = −17.5, d.f. = 240.6, two-sided, Fig. 1a), with a mean value of 79.1% ± 1.1% (mean ± s.e., as below) of air saturation compared to 98.5% ± 0.2% for N_2_. Furthermore, the seasonality in N_2_O saturation was far more pronounced than for N_2_ (Best fitting Generalised Additive Mixed Models, GAMMs, Supplementary Table 1), with a strong minimum for N_2_O in December and maximum saturation in summer (Fig. 1b). Conversely, N_2_ saturation peaked in winter and was lower in spring and summer (Fig. 1c).

The concentrations of dissolved inorganic nutrients (nitrite, NO_2_^−^; nitrate, NO_3_^−^; ammonium, NH_4_^+^, and soluble reactive phosphorus, SRP) were low in the ponds, with NO_2_^−^, NO_3_^−^ and NH_4_^+^often at or below the limit of detection. The concentration of total inorganic nitrogen (TIN as the sum of NO_2_^−^, NO_3_^−^ and NH_4_^+^) was 0.85 ± 0.03 µM across all sampling months (Fig. 1f). SRP concentrations were 0.14 ± 0.01 µM, on average, and, at 5 to 1, the median N to P ratio was markedly lower than Redfield^45^ (16 to 1), indicating primary production in the ponds to be N limited (Fig. 1g). As the ponds were N-limited, primary production must be sustained largely by N fixation (and any unknown atmospheric N deposition), which may have resulted in the undersaturation of N_2_ and N_2_O in the ponds.

Interestingly, N_2_O saturation increased with water temperature (*p* < 0.001, Fig. 1d), suggesting relatively higher net reduction of N_2_O in the cold (*see* Supplementary Fig. 1 for concentration data). Whereas N_2_ saturation showed the opposite pattern, with relatively more net N_2_ reduction at higher temperatures (*p* < 0.001, Fig. 1e) in spring and summer. Moreover, the saturation of dissolved O_2_ in the ponds (at the same depth where the samples for N_2_ and N_2_O were collected) was generally around air-equilibration (104.8% ± 1.8%, median 99.6%), with N_2_ saturation decreasing at higher O_2_ saturations, while N_2_O saturation increased with higher O_2_ saturation (Supplementary Fig. 2). Oxygen saturation was positively correlated with temperature (Supplementary Fig. 2c), probably due to higher temperatures in spring and summer promoting primary production. Therefore, maximum N_2_ undersaturation was probably related to higher primary production in spring and summer^40^. The negative and positive correlations between N_2_ or N_2_O and O_2_ respectively, indicated different controls for the reduction of N_2_ and N_2_O.

### N_2_O and N_2_ fixation by biomass in the ponds

**Fig. 2:**
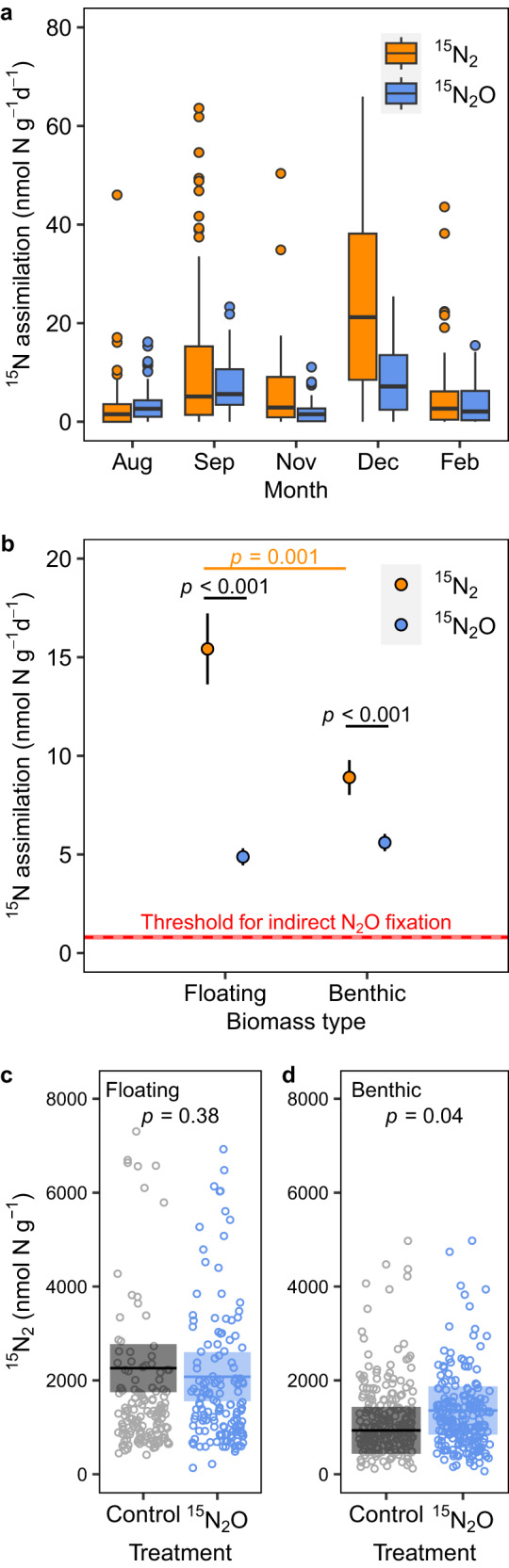
Evidence for the direct assimilation of ^15^N into biomass from either ^15^N_2_ or ^15^N_2_O. ^15^N assimilated into biomass from either ^15^N_2_ or ^15^N_2_O in different months. **a** Each box shows the 25th to 75th percentiles, horizontal lines the median, open circles denote outliers, and whiskers extend to 1.5 times the interquartile range. **b** Rates of ^15^N_2_ assimilation were higher than for ^15^N_2_O assimilation in both biomass types. The red dashed line marks the upper threshold for indirect ^15^N assimilation if ^15^N_2_O was first reduced to ^15^N_2_ prior to assimilation (see Table 1), red shaded area 95% C.I. Data plotted are means ± s.e. Yellow: difference between floating and benthic biomass with ^15^N_2_ (*p* = 0.001, two-sided)_,_ black: difference between ^15^N_2_ and ^15^N_2_O in floating or benthic biomass (*p* < 0.001, two-sided). To exclude extreme outliers the data plotted in **a** and **b** are 95% of the dataset (2.5–97.5% percentiles). Data in **a** and **b** were collected in 5 calendar months for 2 biomass types from 8 to 10 ponds per sampling date (*n* = 303 and *n* = 322 incubations for ^15^N_2_ and ^15^N_2_O treatments, respectively). **c** No significant production of ^15^N_2_ from ^15^N_2_O in the floating biomass, but **d** significant production of ^15^N_2_ in the benthic biomass (*p* = 0.04, two-sided). The horizontal line is the mean and shaded area 95% C.I. (*n* = 351 incubations for both controls and ^15^N_2_O treatments).

To rationalise the undersaturation in both N_2_O and N_2_ in our ponds, we measured fixation of either ^15^N_2_O or ^15^N_2_ (at a range of temperatures, *see* below) by biomass collected from the ponds (Supplementary Fig. 3). We found ^15^N assimilated into biomass from either ^15^N_2_ or ^15^N_2_O in the majority of our incubations (87%, 572 out of 658 incubations, Fig. 2a), with higher rates of ^15^N assimilation with ^15^N_2_ than for ^15^N_2_O with both floating and benthic biomass (*p* < 0.001, *t* = 6.5, d.f. = 369.7, two-sided, Fig. 2b). On average, 11.5 ± 0.9 and 5.3 ± 0.3 nmol g^−1^ d^−1^ (mean ± s.e.) of ^15^N were assimilated into biomass with either ^15^N_2_ or ^15^N_2_O, respectively (Fig. 2b). The rate of ^15^N_2_ assimilation was higher in the floating than the benthic biomass (*p* = 0.001, *t* = 3.3, d.f. = 179.4, two-sided), while ^15^N_2_O assimilation was consistent between the two biomass types (*p* = 0.24, *t* = −1.2, d.f. = 319.1, two-sided).

To distinguish direct N_2_O fixation (N_2_O → NH_4_^+^, Eq. 1) from indirect fixation i.e., that after an initial reduction of N_2_O to N_2_ ([N_2_O → N_2_] N_2_ → NH_4_^+^, Eq. 2) through denitrification, we first checked for any production of ^15^N_2_ from ^15^N_2_O. Overall, the production of ^15^N_2_ in the ^15^N_2_O treatments was not significant in the floating biomass incubations (Fig. 2c), though ^15^N assimilation from ^15^N_2_O was significant (Fig. 2b). In contrast, 30% of the benthic incubations showed measurable ^15^N_2_ production (*p* = 0.04, two-sided, Fig. 2c and Supplementary Fig. 5) but with comparable ^15^N assimilation from ^15^N_2_O (Fig. 2b). Some denitrification is expected given the sediments recognised capacity to consume oxygen^39^, and our 12 h/12 h light/dark incubation-cycle generated oxygen minima overnight that likely facilitated the reduction of N_2_O to N_2_ via denitrification.

In addition, we also compared rates of assimilation against a theoretical upper threshold for indirect assimilation of ^15^N_2_O after reduction to ^15^N_2_ (Table 1 and Fig. 2b). For example, any ^15^N_2_ from the reduction of ^15^N_2_O would be assimilated in proportion to the ^15^N-labelling of the total N_2_ pool, which would be predominantly ambient ^14^N_2_ (Table 1). As a result, any indirect assimilation of ^15^N from ^15^N_2_O should have been ~14-fold lower than what we measured in the incubations where we added ^15^N_2_ directly e.g. 0.8 nmol N g^−1^ d^−1^ vs. 11.5 nmol N g^−1^ d^−1^ (Table 1). In contrast, we measured far higher rates of 5.3 nmol N g^−1^ d^−1^ with ^15^N_2_O, compared to the upper threshold of 0.8 nmol N g^−1^ d^−1^, on average (0.69 to 0.92 nmol N g^−1^ d^−1^, 95% C.I., Fig. 2b). Such disproportionately high activity suggests direct assimilation of ^15^N from ^15^N_2_O into biomass in our freshwater ponds.

**Table 1:** Rationalising N_2_O assimilation as direct N_2_O fixation

Treatment	Process	Frequency of ^15^N-labelling	^15^N assimilation (nmol N g^−1^ d^−1^)
		Direct F_N2_ and F_N2O_ or indirect F_N2_'	
^15^N_2_	Direct N_2_ fixation	F_N2_ = 0.018 = [^15^9μM/(^15^9 μM + ^14^487 μM)]¨	11.5
^15^N_2_O	Direct N_2_O fixation	F_N2O_ = 0.98 = [^15^9 µM/(^15^9 µM + ^14^0.01 µM)]¨	5.3
^15^N_2_O	*Indirect N_2_O fixation	F_N2_’ = 0.0013 = [^15^0.63 μM /(^15^0.63 + ^14^487 μM)]¨	≤0.8

Ambient background concentrations for ^14^N_2_ and ^14^N_2_O in both our ^15^N_2_ and ^15^N_2_O treatments were ~487 μM and 0.01 µM, respectively. We added both ^15^N_2_ and ^15^N_2_O at 9 µM (>98 atom % ^15^N), resulting in initial ^15^N labelling of the ^15^N_2_ and ^15^N_2_O pools of 0.018 and 0.98 (F_N2_ and F_N2O_, respectively). If ^15^N_2_O assimilation was indirect, and ^15^N_2_O was first reduced to ^15^N_2_, then at most 0.63 μM ^15^N_2_ would have been produced and F_N2_’ would have been ≤0.0013. Accordingly, the absolute upper threshold for indirect ^15^N_2_O fixation – in proportion to that directly with ^15^N_2_ (F_N2_) – would have been 0.8 i.e., [(0.0013/0.018) × 11.5] nmol N g^−1^ d^−1^, which is far lower than our measured rates for ^15^N_2_O assimilation (5.3 nmol N g^−1^ d^−1^, on average, Fig. 2b).

*With the predicted maximum ^15^N-labelling of the N_2_ pool (F_N2_’) resulting from the maximum reduction of ^15^N_2_O to ^15^N_2_.

¨Where ^15^ and ^14^ denote the ^15^N and ^14^N species, respectively.

Here we added ^15^N_2_O to our incubations at concentrations many times higher than atmospheric equilibration (9 µM vs. 0.01 μM) and our rates of ^15^N_2_O assimilation are likely upper-potentials. We also characterised the kinetic effect of N_2_O concentration on total N_2_O reduction from 9.2 nM (atmospheric equilibration) to 20,000 nM (Supplementary Fig. 6), which enabled us to estimate N_2_O reduction by biomass at in situ concentrations in the ponds. We then scaled these in situ N_2_O reduction estimates by the amount of benthic biomass in the ponds and compared them to our estimates of N_2_O flux into the ponds calculated using our measurements of N_2_O saturation (Fig. 1 and *see* Supplementary Text 2). Accordingly, we estimated in situ N_2_O reduction by the benthic biomass to be −0.75 µmol N_2_O m^−2^ d^−1^ (Supplementary Text 2) which is equivalent to 56% of the N_2_O flux into the ponds of −1.33 µmol N_2_O m^−2^ d^−1^, on average (range of −3.65 to 0.02 µmol N_2_O m^−2^ d^−1^, including low emissions to the atmosphere in summer). The remaining ~44% of the N_2_O flux is probably driven by microbes associated with the floating biomass (Fig. 2b) or free-living in the water column^42^ and we are confident that our laboratory biomass incubations can rationalise the undersaturation in N_2_O we measured in our ponds. In addition, N_2_ flux into the ponds was −3,934 µmol N_2_ m^−2^ d^−1^, on average (Supplementary Text 2).

### Multiple fates for total ^15^N_2_O reduction

Apart from ^15^N_2_O being assimilated into biomass and the fraction reduced to ^15^N_2_ (above), some fixed ^15^N_2_O as ^15^NH_4_^+^ could potentially “leak” into the pond-water medium to, in turn, be nitrified to ^15^NO_2_^−^ and ^15^NO_3_^−^ (together ^15^NO_*x*_^−^) – all of which comprise total ^15^N_2_O reduction. We characterised total ^15^N_2_O reduction and the proportions of the different end-products and found significant ^15^N_2_O reduction in the majority (289 out of 372 incubations, 78%) of our incubations enriched with ^15^N_2_O. The mean rate of total ^15^N_2_O reduction was 364 ± 27 nmol N g^−1^ d^−1^, with the highest rate of total ^15^N_2_O reduction occurring in December for both floating (850 ± 178 nmol N g^−1^ d^−1^) and benthic biomass (784 ± 158 nmol N g^−1^ d^−1^).

**Fig. 3:**
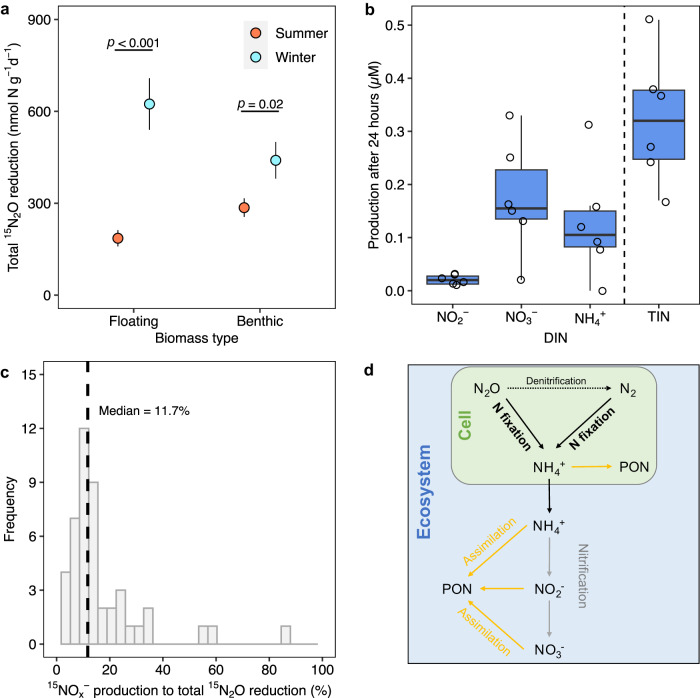
The fate of total ^15^N_2_O reduction in biomass. **a** Total ^15^N_2_O reduction was highest in winter compared to summer in both floating (*p* < 0.001, two-sided) and benthic biomass (*p* = 0.02, two-sided). Data plotted are means ± s.e. from 95% (2.5% to 97.5% percentiles) of the dataset for summer and winter where *n* = 180 and *n* = 160 incubations in summer and winter, respectively (two months for summer, three months for winter). **b** Production of dissolved inorganic nitrogen in ^15^N_2_O incubations. TIN: Total inorganic nitrogen. *n* = 6 incubations for biomass from 6 ponds. Each box shows the 25th to 75th percentiles, horizontal lines the median, and whiskers extend 1.5 times the interquartile range. **c** Distribution of the ratio of ^15^NO_*x*_^−^ production to total ^15^N_2_O reduction, dashed line is the median. Data in **c** are from December, 2020, when the highest rate of total ^15^N_2_O reduction was measured (*n* = 47 incubation bottles, 5 temperatures with 2 biomass types). **d** Simplified diagram showing possible pathways for N_2_O reduction in relation to canonical N_2_ fixation.

To compare summer to winter, we pooled data from November, February and December, for winter, and August and September for summer. Total ^15^N_2_O reduction was highest in winter at 507 ± 49 nmol N g^−1^ d^−1^, compared to 237 ± 21 nmol N g^−1^ d^−1^ in summer, on average (*p* < 0.001, *t* = −5.1, d.f. = 213.4, two-sided) in both floating (*p* < 0.001, *t* = −5, d.f. = 68.6, two-sided) and benthic (*p* = 0.02, *t* = −2.3, d.f. = 148.5, two-sided) biomass (Fig. 3a). The patterns in total ^15^N_2_O reduction measured in the incubations agreed with the seasonal pattern of N_2_O saturation in the ponds (Fig. 1): overall, N_2_O was consumed in both seasons and the ponds were net sinks for N_2_O, with higher N_2_O reduction in winter, corresponding to greater undersaturation in N_2_O in winter.

To test whether N_2_O initially fixed intracellularly as NH_4_^+^ (Eq. 1) could leak into the water i.e., to be available to the wider ecosystem, we performed additional incubations with samples for nutrient measurements (without formaldehyde, *see* methods). Although the concentration of NH_4_^+^ was often below the limit of detection for the colorimetric assay (~0.2 μM), the stronger signal for NH_4_^+^ with N_2_O (*p* = 0.03) indicated some N_2_O fixed as NH_4_^+^ could “leak”. The concentration of total inorganic nitrogen (TIN) was, on average, 0.32 μM higher in incubations enriched with N_2_O than the controls (*p* = 0.001, Fig. 3b). We also characterised the production of ^15^NO_*x*_^−^ from ^15^N_2_O in December, 2020 (winter), when rates of total ^15^N_2_O reduction were highest. The rate of ^15^NO_*x*_^−^ production was 280 ± 46 nmol g^−1^ d^−1^ (mean ± s.e.), which accounted for 11.7% (median) of total ^15^N_2_O reduction (Fig. 3c). Together, these results show that some N_2_O fixed as NH_4_^+^ can be lost to the water and further oxidised to NO_*x*_^−^ through nitrification both of which could be assimilated into PON by the wider community (Fig. 3d).

### The temperature dependence of N_2_O fixation

**Fig. 4:**
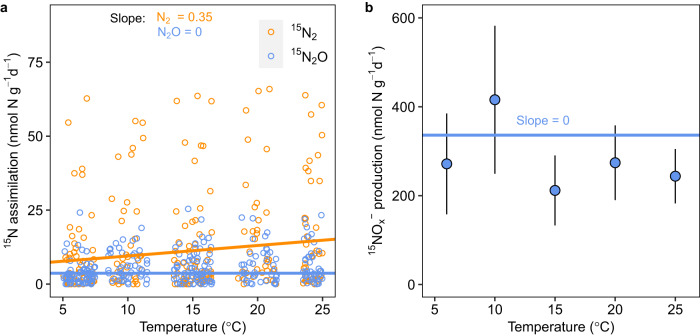
Temperature sensitivity of ^15^N assimilation from ^15^N_2_ and ^15^N_2_O and ^15^NO_*x*_^−^ production from ^15^N_2_O. **a** Temperature sensitivities for ^15^N assimilation from ^15^N_2_ and ^15^N_2_O were different (*p* = 0.025, two-sided), increasing at higher temperatures for ^15^N_2_ (slope: 0.35, 95% CI: 0.12–0.5) but ^15^N_2_O. As the data were skewed, we used median regression models to minimise bias from outliers. The regression in **a**, uses the whole dataset but only 95% of the dataset (2.5% to 97.5% percentiles) are presented (*n* = 303 and *n* = 322 for ^15^N_2_ and ^15^N_2_O, respectively, for 5 months, 2 biomass types). **b** similarly, ^15^NO_*x*_^−^ production from ^15^N_2_O was also invariant to temperature. Data in **b** are from December, 2020, when total ^15^N_2_O reduction was highest (*n* = 47 incubations, 5 temperatures, 2 biomass types), blue line is a first-order linear regression, mean ± s.e. As the temperature sensitivity of ^15^N assimilation and ^15^NO_*x*_^−^ production was consistent between floating and benthic biomass, the data in **a** and **b** have been pooled for both biomass types.

As seasonal changes in temperature drove contrasting patterns in N_2_O and N_2_ saturation, we characterised the effect of temperature on N_2_O and N_2_ reduction by incubating biomass from the ponds at temperatures from 6°C to 25°C. Assimilation of ^15^N from ^15^N_2_ increased at higher temperatures (*p* = 0.005, *t* = 2.8, d.f. = 301, Fig. 4a), with an estimated Q_10_ of 1.38. In contrast, assimilation of ^15^N from ^15^N_2_O was consistent across all temperatures with no discernible temperature sensitivity. The large variance in Fig. 4a may in part be due to simply normalising the ^15^N assimilation data to a unit of dry biomass in each incubation, whereas the communities responsible for N_2_ or N_2_O assimilation could be heterogeneous in the biomass samples and across different months of the year. In addition, rates of ^15^NO_*x*_^−^ production from ^15^N_2_O were also consistent across incubation temperatures (Fig. 4b), which, again, suggested that N_2_O fixation is not sensitive to temperature (i.e., Fig. 4a, b).

### *nifH* communities in relation to N_2_O reduction

The fact that here N_2_O fixation appears less sensitive to temperature than N_2_ fixation supported our hypothesis that fixing N_2_O is less energy demanding than fixing N_2_. Here we aimed to address our question of whether N_2_O fixation is mediated by the whole N_2_ fixing community or a sub-set, using a long-term incubation with N_2_O-enriched biomass.

**Fig. 5:**
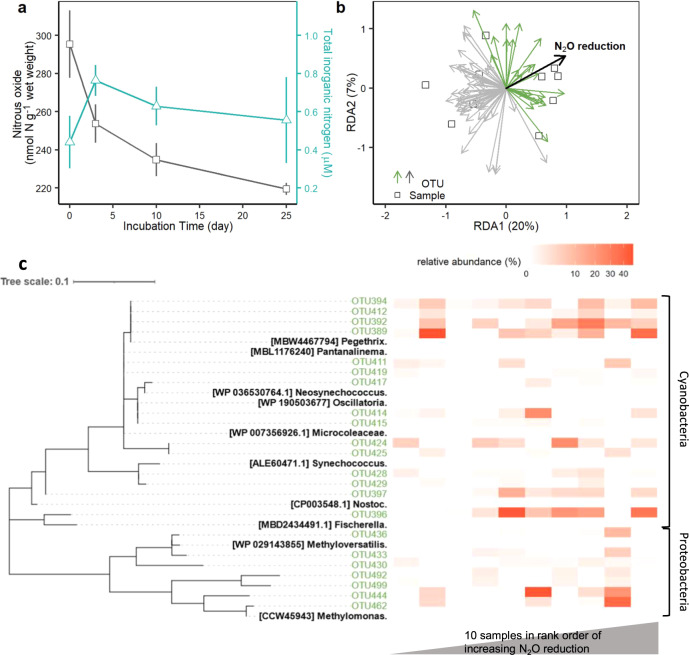
Biomass *nifH* community in relation to N_2_O reduction. **a** Biomass incubated for 25 days reduced 76 nmol N_2_O-N g^−1^ (dry weight) on average (*n* = 40 incubations for biomass enriched with N_2_O) with activity peaking before inorganic nitrogen accumulated. Data plotted are means ± s.e. **b** Redundancy analysis (RDA) revealed positive correlations between the initial rates of N_2_O reduction and the relative abundance of 22 *nifH* OTUs, including 15 Cyanobacterial OTUs and 7 Proteobacterial OTUs (arrows in green). **c** Heat-map (white to dark red) of the relative abundance of the 22 *nifH* OTUs (in green), identified in **b**, in samples (columns, *n* = 10) in rank order of increasing rate of N_2_O reduction. *n* = 10 in **b** and **c**. N_2_O reduction is presented as a black arrow in **b** and as a grey ascending triangle in **c**.

N_2_O reduction was most rapid during the first 3 days of the incubation and started to decline after the increase in total inorganic nitrogen (TIN, sum of NO_3_^−^, NO_2_^−^ and NH_4_^+^) from 0.44 µM to 0.76 µM and (Fig. 5a and Supplementary Fig. 7a). We terminated the incubation after 25 days when oxygen production from photosynthesis started to decline (Fig. 5a and Supplementary Fig. 8b) and characterised the abundance and structure of the *nifH* community.

We first tested whether N_2_O reduction was related to the abundance of the whole *nifH* community (copy numbers of *nifH* per g wet biomass) but found no relationship (*p* = 0.21, *F* = 1.74, d.f.=18). We then tested whether N_2_O reduction was related to a sub-community by looking for any changes in diversity or composition of the *nifH* community over the 25-day incubation. Our primers amplified 894 well-represented OTUs ( > 20 reads in at least 3 samples) of which only 227 were identified as *nifH* OTUs (*see* Methods and Supplementary Figs. 9 and 10). However, neither the diversity (*p* = 0.81, *t* = 0.24, d.f. = 17.5, two-sided *t*-statistic tested on the means of Shannon index) nor the composition (*p* = 0.99, PERMANOVA, *see* also Supplementary Fig. 11 for unchanged *nifH* community composition at 3, 10 and 25 days) of the overall *nifH* community changed significantly during the 25-day incubation.

As an alternative, we used redundancy analysis (RDA) to ordinate the relative abundances of *nifH* OTUs and the initial rates (i.e., in 10 samples up to day 3) of N_2_O reduction to identify any likely N_2_O fixing candidates (Fig. 5b). Since N_2_O reduction may be mediated by a subset of the whole *nifH* community, we relaxed our definition of a well-represented OTU to include <20 reads in at least 3 samples (see Supplementary Fig. 9) which retained 72 out of 227 *nifH* OTUs. Of those 72 OTUs, the relative abundance of 22 were positively correlated with N_2_O reduction i.e., in the ordination their arrows pointed in a similar direction to the arrow for N_2_O reduction. The positive correlations for the 22 OTUs, including 15 Cyanobacteria and 7 Proteobacteria, were further explored by visualising their relative abundance in each biomass sample in rank order of increasing rate of N_2_O reduction (Fig. 5c). Among the 15 Cyanobacterial OTUs, *Pegethrix*-like OTU392 and OTU394 (100% identical protein sequence to *Pegethrix*) and the *Fischerella*-like (>99% identical) OTU396 appeared to not only be more common, but they were also more strongly correlated with the initial rates of N_2_O reduction. While OTU412 and OTU389 were also identical to *Pegethrix* they were either relatively rare or less-well correlated, respectively. Despite the two *Methylomonas*-like (>99 % identical) Proteobacterial OTU444 and OTU462 being less common than the Cyanobacterial candidates, exisiting in only four samples, their higher relative abundances coincided with higher rates of N_2_O reduction. Moreover, combinations of the five strongest (OTUs 392, 394, 396, 444, 462) N_2_O fixing candidates were not only present in the ten samples used to determine the initial rates of N_2_O reduction but all 40 samples enriched with N_2_O for our 25-day incubation (Supplementary Fig. 9).

## Discussion

In ecosystems with limited fixed nitrogen (e.g. inorganic NO_2_^−^, NO_3_^−^, NH_4_^+^), primary production is tightly coupled to N-fixation – typically recognised to be N_2_ gas (Eq. 2). The undersaturation in N_2_O reported here means that the reduction of N_2_O was greater than its rate of delivery either from the atmosphere or biological sources in the ponds, which shows that these N-limited ponds were overall sinks for N_2_O, including direct N_2_O fixation (Eq. 1).

Others have argued for direct N_2_O fixation on the premise that if N_2_ production was not detected in incubations with N_2_O, then N_2_O fixation was direct^5,30^. Here, besides not detecting ^15^N_2_ production in 76% of our incubations (Fig. 2b), our disproportionate fixation of N from ^15^N_2_O relative to that measured with ^15^N_2_ provides more substantive evidence for direct N_2_O fixation (Table 1). Direct N_2_O fixation represents an alternative to the only widely recognised sink for N_2_O – namely denitrification^6,29^. In addition, our estimation of in situ N_2_O fixation helps to rationalise the undersaturation and resultant flux of N_2_O into our ponds (see Supplementary text 2). Further, as the scale of N_2_O undersaturation in our ponds (Fig. 1a) is in line with many other studies also reporting undersaturation in N_2_O in freshwaters (typically ~70–100%)^10,16–18,21^, this indicates that direct N_2_O fixation could explain the unaccounted for N_2_O undersaturation in many freshwaters^8–10,16–21^.

We can see similar seasonal trends to what we report here in previous accounts of N_2_O undersaturation. For example, boreal lakes, ponds and rivers show undersaturation in N_2_O which is strongest at coldest temperatures^19^. In Boreal peatlands, N_2_O was undersaturated mostly in spring, increasing to maximum oversaturation in summer, then decreasing to near equilibrium in autumn^46^. From the same study, soils acted as net N_2_O sources at higher temperatures, while most N_2_O sinks occurred below 13 °C^46^. In the surface waters of the Baltic Sea, N_2_O was most undersaturated in winter (December), but was oversaturated in summer and autumn^11^. However, these studies generally lacked a clear explanation for the occurrence and the temperature dependence of N_2_O undersaturation, whereas we now offer an explanation.

Our findings demonstrate different temperature dependencies for N_2_ and N_2_O fixation. This difference in N_2_ versus N_2_O is supported not only by the opposing seasonal patterns in N_2_ and N_2_O saturation in our ponds, but also by the experimentally determined different temperature sensitivities for the assimilation of N_2_ and N_2_O by biomass in our incubations. Moreover, the results from our incubations support the seasonal patterns in N_2_ and N_2_O saturation in the ponds – with the higher rates of N_2_O reduction in incubations in winter than in summer, matching the stronger N_2_O undersaturation in the ponds in winter, and the elevated temperature effect on N_2_ assimilation agrees with that for N_2_ saturation in our ponds. Here the apparent lack of temperature sensitivity of N_2_O fixation (Fig. 4) suggests that the N-fixing communities may be strongly adapted to substrate limitation (Supplementary Fig. 6), with dissolved N_2_O typically at ~10 nM in the ponds compared to ~490 µM for N_2_. This strong kinetic effect of substrate availability on N_2_O fixation has also been reported in incubations with surface seawater^23^ and pure culture of marine cyanobacteria *Trichodesmium* sp^5^. Similar stronger limitation of activity by substrate over temperature is also recognised in other autotrophs such as the methane oxidising methanotrophs^47,48^.

The contrasting temperature sensitivies of N_2_O and N_2_ fixation are probably associated with the energetic advantage of using N_2_O instead of N_2_ as a N-substrate for N-fixation^49^. Here we compiled data for studies measuring N_2_ fixation in both aquatic and terrestrial ecosystems (Supplementary Fig. 12 and the references cited therein) which clearly shows N_2_ fixation increases at higher temperatures. On the other hand, as dissociating the N bond in N_2_O only requires about half of the energy compared to N_2_ (refs. ^49,50^), N_2_O should be easier to fix at colder temperatures and a higher proportion of total N fixation could be dependent on N_2_O in the cold. For example, with our pond biomass the fraction of total N-fixation coupled to N_2_O at 6°C was 26% higher than that at 25°C (Fig. 4a). This energy saving offered by fixing N_2_O in the cold might explain why N_2_O undersaturation in our ponds was strongest during the colder months and may also explain the undersaturation reported in cold, boreal environments and Baltic Sea^11,19,46^.

In addition, in northern latitudes cold temperatures typically occur alongside lower light and the ~18% energy saving from fixing N_2_O (Eq. 1) compared to N_2_ (Eq. 2) could provide an over-wintering strategy for a subset of the photosynthetic community. Besides photosynthesis, some chemosynthetic microbes could also benefit. For example, some *Methylomonas* spp. are known to fix N_2_ and were identified here amongst our potential N_2_O fixing candiates. Some 32% of the energy yielded from oxidising CH_4_ to CO_2_ can be expended on assimilating CH_4_ into biomass (equation S6, Supplementary Text 1) with another 56% needed to fix N_2_ compared to 47% for N_2_O. Given that methane concentrations are lowest in our ponds in winter^39^ - and more widely methane production is known to be tightly coupled to primary production in spring and summer^51^ - fixing N_2_O could offer an advantage over N_2_ when resources are limited.

The concept that N_2_ fixation in general is routinely supressed by inorganic N (>~1 µM) has been revised^52^. For example, while there are numerous examples of N_2_-fixing activity being suppressed by inorganic nitrogen in pure cultures of *Trichodesmium* spp., others have reported its activity in euphotic ocean waters (whole water and *Trichodesmium* spp. isolates) with ~5 µM to 21 µM NO_3_^−^ (refs. ^52,53^), which may indicate short-term tolerance to NO_3_^−^. In contrast, the N-fixing community in our ponds is exposed to chronic, year round inorganic nitrogen starvation (i.e., <1 µM). Our 25-day incubation showed N_2_O reduction declined after an increase in inorganic nitrogen (NO_3_^−^ and NH_4_^+^) to ~0.8 µM (Fig. 5a), indicating a low-threshold concentration for inorganic nitrogen that apparently limits N_2_O fixation. Such a threshold also reflects the co-occurance of N_2_O undersaturation (Fig. 1a) with <1 µM inorganic nitrogen throughout the year in our ponds (Fig. 1f). In contrast, N-rich ecosystems generally act as N_2_O sources^8,25^, while N_2_O sinks - mediated through N_2_O fixation – are likely to be found in pristine, cold ecosystems^19,21,22,26–28^.

To date, it is not clear which microorganisms are responsible for N_2_O fixation in natural ecosystems. A few studies have reported N_2_O fixation in sea water^5,23^ and soybean root nodules^30^, but only one study, on pure cultures of the marine cyanobacteria *Trichodesmium* sp. and *Crocosphaera* sp., has related the *nifH* gene to N_2_O fixation^5^. Since being set up in 2005, our nitrogen-limited ponds have developed diverse diazotroph communities^42^, comparable to those in estuaries^54^, freshwater^55^ and seawater^56^. Here we set out to link cause to effect by attempting to enrich N_2_O fixing candidates but failed to detect any changes in the total *nifH* community after 25 days enrichment with N_2_O (Supplementary Fig. 11). This might have been due to the decline in rate of N_2_O reduction, as a result of the parallel accumulation in inorganic nitrogen, or longer incubations being needed to detect any change in the total *nifH* community that is likely slow-growing. In our ordination analysis, five OTUs emerged as the strongest potential candidates for N_2_O fixation i.e., their relative abundance is at least correlated with N_2_O reduction activity. Of these, one is identical to *Fischerella*, a common diazotroph in freshwater^55^ and seawater^57^ and also identified in seawater undersaturated in N_2_O^23^ and another two being identical to *Pegethrix*^58^, a newly identified genera of filamentous Cyanobacteria. In addition, proteobacterial methanotrophic diazotrophs have been related to N-fixing activity in N-limited freshwaters^59^, and relatives of our two *Methylomonas*-like candidates are known to grow on N_2_ as their sole nitrogen source^60^. Whether or not our candidate N_2_O-fixers are responsible for the widely reported undersaturation in N_2_O in natural waters needs characterising directly but our first attempt here at least suggests N_2_O fixation is likely mediated by a subset of diazotrophic communities.

In addition, our work shows that N_2_O fixation can occur in an abundance of N_2_ i.e., against the high natural N_2_ background. This indicates that N_2_O fixation could happen in natural ecosystems replete in N_2_ and provides further insight into the communities responsible for N_2_O fixation. For example, *nifH* communities could fix N_2_ and N_2_O randomly, with the ratio of N_2_O to N_2_ fixation being simply proportional to the relative availability of N_2_O to N_2_. However, the distinct seasonal patterns we measured for N_2_ and N_2_O undersaturation (Fig. 1), coupled to disproportionate rates of N_2_O fixation (Fig. 2) and higher proportion of N_2_O fixation at colder temperatures (Fig. 4a) - all indicate that a specialised subset of the *nifH* community (Fig. 5) likely favoured N_2_O over N_2_ at colder temperatures in support of our *nifH* ordination analysis.

To put our estimates of N fixation into an ecological context, we compared estimates of the N_2_ flux (Supplementary Text 2) with former estimates of gross primary production (GPP) in the ponds^40^. For example, the average net N_2_ flux into the ponds was 3934 µmol N_2_ m^−2^ d^−1^, which, assuming Redfield ratios of 106:16 for C:N, could sustain GPP of 52,126 µmol C m^−2^ d^−1^ and which is comparable to GPP measured previously of 51,488 to 70,792 µmol C m^−2^ d^−1^ (ref. ^40^). Moreover, the seasonal dynamics in N_2_ flux in our study also matched that of GPP reported previously^40^, with both peaking in the summer (Fig. 1b). In contrast, while the flux of N_2_O is comparatively minor (~0.03 %) in terms of supporting GPP, it is great enough to maintain a strong sink for N_2_O.

To date, denitrification in either anoxic or oxic-to-anoxic transitioning waters is still the only widely recognised sink for N_2_O^6,7,29^. Here, as an alternative to denitrification, direct N_2_O fixation can rationalise the undersaturation in N_2_O in our ponds and could also explain the various unaccounted for N_2_O sinks – of similar magnitude – reported in natural, pristine waters^10,17,19,21,22,28,46^. As N_2_O undersaturation is favoured in the cold, rising temperatures could erode this natural sink for such a potent climate-gas.

## Methods

### Nutrient analysis

Temperature and O_2_ were measured in each pond using HQD portable metre (Hach). Samples of water for nutrient analysis were filtered (0.45 μm PES, 25 mm, pre-washed with deionized water) into Falcon tubes, kept cool and frozen at −20 °C back in the laboratory. Samples were thawed overnight at 4 °C and analysed by standard wet-chemistries for NO_2_^−^, NO_3_^−^, NH_4_^+^ and SRP on an autoanalyzer (San^++^, SKALAR Analytical B.V.)^61^ against certified reference materials, traceable to NIST. The limits of detection were 0.05 μM and 0.1 μM for NO_2_^−^ and NO_*x*_^−^ (NO_2_^−^ + NO_3_^−^), respectively, 0.2 μM for NH_4_^+^ and 0.05 μM for SRP. SRP and total inorganic nitrogen (TIN, NO_3_^−^ + NO_2_^−^ + NH_4_^+^) below detection limits were omitted from any calculations.

### Dissolved N_2_ and N_2_O in the ponds

For dissolved N_2_ and N_2_O analyses, water samples were taken carefully at mid-water-depth (~20 cm from the surface) from each pond using a 60 mL syringe and tubing. Five gas-tight vials (12 mL Exetainer, Labco, two vials for N_2_O and three for N_2_) for each pond were allowed to overflow three times, preserved with ZnCl_2_ (50 μL of 50% w/v)^62^, closed and mixed by hand. Extra pond water samples for reference N_2_ saturation were collected and preserved along with samples of air.

In a temperature-controlled laboratory at 22 °C, references for N_2_ saturation were prepared by equilibrating the pond water with the laboratory air, and then water and air samples were collected as for the field samples. Helium headspaces (2 mL 99.999% purity) were created in all sample and reference vials, followed by 24 h equilibration on an orbital shaker (SSL1, Stuart) in the same laboratory and all vials weighed to determine the exact volume of headspace and water.

For N_2_O, 100 μL of sample headspace was injected by an autosampler into a gas chromatograph fitted with a µECD (Agilent Technology UK Ltd., South Queensferry, UK) along with air samples using conditions described previously^63^. Calibration was performed against known concentrations of N_2_O from a NOAA standard (traceable to the SI unit “amount of substance fraction”) at 359.73 ppb or 120 ppb and 1.04 ppm and 96 ppm from BOC, UK, cross-calibrated to the NOAA standard. The precision for N_2_O concentration was 2% (coefficient of variation, *n* = 10). The total concentration of N_2_O in each vial was calculated using solubility coefficents^64^ as described before^63^ and the degree of over- or under-saturation calculated by comparison to the expected concentration of N_2_O for pond water at equilibrium with the atmosphere (see Supplementary Fig. 1).

For N_2_ analysis, we used the published N_2_:Ar method^65^. 100 μL of headspace was injected by an autosampler into an elemental analyzer, (Flash EA 1112 series, Thermo Finnigan) to remove O_2_ by the hot-copper reduction, before passing to a continuous flow isotope ratio mass spectrometer (CF-IRMS, Delta V Plus, Thermo Finnigan). Throughout each run, air samples were analysed to correct for drift and the expected concentrations for N_2_ or Ar in the headspace (C_hs_) calculated using the solubility of N_2_ and Ar for air at both field (K_field_) and laboratory (K_lab_) temperature^66^:

4 $${{{\mbox{C}}}}_{{{\mbox{hs}}}}\times {{{\mbox{V}}}}_{{{\mbox{hs}}}}+{{{\mbox{C}}}}_{{{\mbox{hs}}}}\times {K}_{{{{{{\rm{lab}}}}}}}\times {{{\mbox{V}}}}_{{{\mbox{aq}}}}={{{\mbox{C}}}}_{{{\mbox{field}}}}\times {{{{\mbox{K}}}}_{{{\mbox{field}}}}\times {{\mbox{V}}}}_{{{\mbox{aq}}}}$$

Where V_hs_ and V_aq_ are the volumes of headspace and water in a vial and C_hs_ and C_field_ the concentration of either gas in the headspace or field air, respectively. The saturation of N_2_ in the samples was then derived by comparing the measured to expected ratio of N_2_ to Ar in the samples to that in the pond water ref. ^67^:

5 $${{{\mbox{N}}}}_{2}{{\mbox{Saturation}}}(\%)={\left(\frac{{{{\mbox{N}}}}_{2}/{{\mbox{Ar}}}\, {{\mbox{measured}}}}{{{{\mbox{N}}}}_{2}/{{\mbox{Ar}}}\, {{\mbox{expected}}}}\right)}_{{{\mbox{Sample}}}}\bigg/{\left(\frac{{{{\mbox{N}}}}_{2}/{{\mbox{Ar}}}\, {{\mbox{measured}}}}{{{{\mbox{N}}}}_{2}/{{\mbox{Ar}}}\, {{\mbox{expected}}}}\right)}_{{{\mbox{reference}}}}\times 100$$

Precision for the ratio of N_2_ to Ar for triplicate reference water and air standards was 0.1% and 0.05% (coefficient of variation), respectively. We also tested the effect of calculating N_2_ saturation with different references, with the ratio for deionized versus pond water being 99.7% and 99.82%, on average (*n* = 20 and *n* = 8, respectively).

### Biomass incubations to characterise N_2_ and N_2_O fixation

Two types of biomass were collected from the ponds for the routine (24-h) incubations (Supplementary Fig. 3) and see below for the 25-day incubation. Floating assemblages on the ponds, comprising *Oedogonium* spp. and microorganisms attached to the filaments (Supplementary Fig. 3b) and green or yellow benthos assemblages (Supplementary Fig. 3c), sampled avoiding the sandy sediments beneath. Samples of biomass were collected in sterile Falcon tubes (50 mL) and transported back to the laboratory in a cool box and stored overnight at 15 °C before preparing the incubations. Despite the ponds being low in dissolved inorganic nitrogen, we standardised nutrient concentrations in the incubations by using an artificial pond water medium devoid of fixed N comprising: CaCl_2_ (0.5 mM), KCl (1 mM), MgSO_4_ (0.25 mM), KHCO_3_ (0.7 mM) and NaHCO_3_ (0.5 mM) in deionized water. P was added to 0.08 μM of NaPO_4_, based on measured SRP concentrations in the ponds.

### Incubations of biomass with ^15^N_2_ and ^15^N_2_O tracers

Floating or benthic biomass was weighed (~3 g wet weight) into 12 mL gas-tight vials and, for the ^15^N_2_O treatment, filled with oxygen-saturated artificial pond water and closed. 100 μL of water was replaced by ^15^N_2_O stock solution (see below) with a gastight syringe. For the ^15^N_2_ treatment, each vial was filled with 10 mL of oxygen-saturated artificial pond water and 2 ml of ^15^N_2_ stock and closed (see below). All incubations were prepared without a headspace to ensure ^15^N-substrate concentrations were the same under different temperatures. Parallel controls were prepared in the same way without either ^15^N-gas.

T_0_ (Time zero) vials were killed with 200 μL of 50% (w/v) formaldehyde immediately and the remainder incubated in temperature-controlled orbital incubators (SI500, Stuart at 50 cycles min^−1^) at 6, 10, 15, 10 and 25 °C on a 12 h light/12 h dark cycle for 24 h. Time final (T_f_) samples were killed as above, brought to 22 °C, helium headspaces created in all vials, and all allowed to equilibrate for 24 h, as above.

### Stock solutions for ^15^N_2_ and ^15^N_2_O additions to the biomass incubations

To avoid the recognised equilibration problems with the ^15^N_2_ “bubble method”, especially during short-term incubations^68^, we first made an aqueous ^15^N_2_ stock with the artificial pond water. 200 mL of artificial media were injected into a 0.5 L gas sampling bag along with 40 mL ^15^N_2_ gas (98% atom % ^15^N, Sigma-Aldrich) and allowed to equilibrate for 24 h while gently rocking. ^15^N_2_O stock solutions were prepared by replacing 3 mL of water with ^15^N_2_O gas (98% atom % ^15^N, Cambridge Isotope Laboratories, Inc.) in a 50 mL sealed serum bottle.

The solubility of N_2_ is low and to maximise ^15^N_2_ labelling we added a relatively large amount (~2 ml) of ^15^N_2_ stock solution to the 12 mL vials (~16% v/v). To keep the dissolved nutrients and gases at background levels in the ^15^N_2_ treatments -and the same as in the controls and ^15^N_2_O treatment- we used artificial freshwater medium instead of deionized water for preparing ^15^N_2_ stocks. In contrast to N_2_, N_2_O is highly soluble, with only ~100 μL of ^15^N_2_O stock being needed in each treatment (~0.8% v/v) to reach the comparable concentration of ^15^N-N_2_ addition (~10 μM). ^15^N_2_O and ^15^N_2_ stocks were prepared fresh before each experiment and their respective dosages tested by spiking controls.

### Characterising total ^15^N_2_O reduction

The concentration of ^15^N_2_O in the samples was measured on a CF-IRMS (Delta V Plus, Thermo-Finnigan) with an automated trace gas pre-concentrator (PreCon, Thermo-Finnigan)^63^. A sub-sample from the headspace of each sample was transferred to a 12 mL air-filled gas-tight vial. The high ^15^N-labelling of ^15^N_2_O (on average, 97.7% of ^46^N_2_O) in the ^15^N_2_O treatment meant only a small aliquot of sample (10 μL) was needed to keep the signal within the measurable range of ^46^N_2_O. Mass-to-charge ratios were measured for *m/z* 44, 45 and 46 and the concentration of N_2_O determined by calibration against known amounts (0.02–2 nmol) of natural abundance N_2_O (96 ppm N_2_O standard, BOC, UK)^63^. Note, whether the mass spectrometer is calibrated with high purity ^15^N_2_O or natural abundance N_2_O, the signal-to-mole ratio is constant (Supplementary Fig. 13). Here the concentration of total ^15^N_2_O is expressed as ^15^N_2_O = ^45^N_2_O + 2 × ^46^N_2_O and the reduction of ^15^N_2_O calculated by subtracting ^15^N_2_O concentrations in T_f_ samples from T_0_ samples.

### Characterising any dissimilatory reduction of ^15^N_2_O to ^15^N_2_

Any production of ^15^N_2_ in the ^15^N_2_O treatments was measured by the CF-IRMS (Delta V Plus, Thermo Finnigan), after bypassing the copper reduction step to avoid reduction of ^15^N_2_O to ^15^N_2_ (ref. ^69^). The concentration of total N_2_ was calculated from the solubility of N_2_ (ref. ^66^) and the signal of total N_2_ mole masses i.e., *m/z* 28, 29 and 30 in the samples and air standards^70^. Drift in *m/z* 30 was corrected by inserting air standards for every 10 samples. Changes in the concentration of ^15^N_2_ (Δ^15^N_2_, nmol N d^−1^) were calculated by the excess ^15^N_2_ in ^15^N_2_O treatments compared to the controls, where Δ^15^N_2_ = Δ^29^N_2_ + 2 x Δ^30^N_2._ The limit of detection for Δ^15^N_2_ in the incubations is ~0.14 μM.

### Characterising assimilation of ^15^N_2_O or ^15^N_2_ into biomass

After all the gas measurements, samples were centrifuged and the supernatants filtered (as above). The remaining biomass was dried, homogenised and sub-samples weighed into tin caps (6 × 4 mm, Elemental Microanalysis) for elemental analysis as described previously^41^. The level of ^15^N enrichment in biomass incubated with either ^15^N_2_ or ^15^N_2_O was then calculated by the difference in excess ^15^N atom % relative to the controls, where excess ^15^N atom % is the difference in ^15^N atom % between T_0_ and T_f_ in the 24-h incubations:

6 $${}^{15}{{{{{\rm{N}}}}}}\,{{{{{\rm{enrichment}}}}}}={({{{{{\rm{excess}}}}}}{}^{15}{{{{{\rm{N}}}}}}\, {{{{{\rm{atom}}}}}}\%)}_{{{{{{\rm{Treatment}}}}}}}-{({{{{{\rm{excess}}}}}}{}^{15}{{{{{\rm{N}}}}}}\, {{{{{\rm{atom}}}}}}\%)}_{{{{{{\rm{Control}}}}}}}$$

Rates of ^15^N assimilation (nmol ^15^N g^−1^ day^−1^) by biomass into particulate organic nitrogen (PON) were calculated as:

7 $${}^{15}{{{{{\rm{N}}}}}}\,{{{{{\rm{assimilation}}}}}}\,{{{{{\rm{rate}}}}}}={{{{{\rm{PON}}}}}}\times {}^{15}{{{{{\rm{N}}}}}}\,{{{{{\rm{enrichment}}}}}}/(\varDelta {{{{{\rm{t}}}}}}\times {{{{{\rm{dw}}}}}})$$

Where PON is particulate organic nitrogen in a sample of biomass, $\Delta {{\mbox{t}}}$ is the incubation time (24 h), and dw the dry weight (g).

### Characterising ^15^N_2_O fixation

As total ^15^N_2_O reduction includes both assimilatory ^15^N_2_O fixation and dissimilatory ^15^N_2_O reduction to ^15^N_2_, total ^15^N_2_O fixation can be calculated by subtracting ^15^N_2_ production from total ^15^N_2_O reduction:

8 $${{{{{\rm{Total}}}}}}{}^{15}{{{{{\rm{N}}}}}}_{2}{{{{{\rm{O}}}}}}\,{{{{{\rm{fixation}}}}}}={{{{{\rm{Total}}}}}}{}^{15}{{{{{\rm{N}}}}}}_{2}{{{{{\rm{O}}}}}}\,{{{{{\rm{reduction}}}}}}-{}^{15}{{{{{\rm{N}}}}}}_{2}\, {{{{{\rm{production}}}}}}$$

Where total ^15^N_2_O fixation includes ^15^N_2_O assimilated into biomass, as well as any fixed ^15^N_2_O present in the pond water medium as dissolved inorganic nitrogen (^15^DIN, e.g., ^15^NH_4_^+^, ^15^NO_2_^−^ and ^15^NO_3_^−^):

9 $${{{{{\rm{Total}}}}}}{}^{15}{{{{{\rm{N}}}}}}_{2}{{{{{\rm{O}}}}}}\,{{{{{\rm{fixation}}}}}}={}^{15}{{{{{\rm{N}}}}}}_{2}{{{{{\rm{O}}}}}}\,{{{{{\rm{assimilation}}}}}}+{}^{15}{{{{{\rm{D}}}}}}{{{{{\rm{IN}}}}}}\,{{{{{\rm{production}}}}}}$$

We characterised any ^15^DIN production coupled ^15^N_2_O fixation by measuring ^15^NO_*x*_^−^ (i.e., ^15^NO_3_^−^ + ^15^NO_2_^−^) with sulfamic acid^62^, testing for any effect of formaldehyde on the ^15^NO_*x*_^−^ assay (Supplementary Fig. 14). Due to the high ^15^N_2_O background, it was not possible to measure any ^15^NH_4_^+^ from ^15^N_2_O using the sensitive sodium-azide assay as it converts ^15^NO_2_^−^ to ^15^N_2_O. Further, as the formaldehyde preservative interferes with the colorimetric NH_4_^+^ assay, we did additional incubations in October 2021 without formaldehyde, following the exact incubation procedure described above. Here, samples for DIN were immediately centrifuged and frozen at −20 °C while parallel samples for gases were treated as above. Concentrations of DIN in controls and ^15^N_2_O treatments were measured by the automated wet-chemistry autoanalyzer (*see* ‘Nutrient analysis’ in Methods), while changes in N_2_O concentrations were measured by GC/µECD^63^.

### *nifH* communities in relation to N_2_O reduction over a 25-day incubation

To characterise any *nifH* communities potentially involved in N_2_O fixation, we incubated floating biomass from the ponds with excess N_2_O for as long as possible, looking either for changes in the *nifH* community or relationships between particular *nifH* families and N_2_O reduction. Floating biomass was collected from 10 ponds in May 2021 (as above) and once back in the laboratory kept in a temperature-controlled room at 15°C overnight. The next day, 7 g wet biomass was transferred into 70 mL serum bottles (*n* = 80, 8 serum bottles per pond), filled with water and sealed. N_2_O stock solution (600 µL as above) was injected into half of the serum bottles, while venting water through a needle, to create an initial N_2_O concentration of ~10 µM and the remaining serum bottles left unamended as controls. Temperature-controlled incubations were carried out on a 12 h:12 h light/dark cycle as above. A total of 20 serum bottles (10 with N_2_O and 10 controls) were sacrificed after 0, 3 and 10 days of incubation, respectively, while the last 20 serum bottles were incubated until the daily maximum in oxygen started to decline. Oxygen was measured with optical sensors (OXSP5, FireSting®, Pyro Science GmbH, Germany) at ~2-hourly intervals after lights and the incubations terminated on day 25 when daily maximum oxygen started to decline (Supplementary Fig. 8).

Sub-samples of water were transferred from each serum bottle into a 3 ml gas-tight vial (Exetainers, Labco) after 0, 3, 10 and 25 days of incubation, fixed with 50 µl formaldehyde, sealed and stored at room temperature. After creating a helium headspace N_2_O concentrations were measured by GC/µECD (as above). The remaining water was filtered and frozen at −20 °C for later quantification of NO_3_^−^, NO_2_^−^ and NH_4_^+^, as above. Biomass was frozen at −20 °C until DNA extraction (June 2021) from ~0.5 g of wet biomass (DNeasy PowerSoil kit, Qiagen) as per the manufacturer’s instructions.

### *nifH* gene abundance (qPCR) and library preparation

Gene abundance of *nifH* was determined using qPCR with IGK3/DVV (forward, 5’-GCIWTHTAYGGIAARGGIGGIATHGGIAA-3’; reverse, 5’- ATIGCRAAICCICCRCAIACIACRTC-3’)^71^ using a CFX384 Touch Real-Time PCR (Bio-Rad) in 10 µL reactions containing 5 µL SensiFAST SYBR No-ROX mastermix (Meridian Bioscience), 0.8 µL of each primer (10 µM), 0.8 µL DNA template and 2.6 µL molecular biology quality water (MBQW). The qPCR programme was 98 °C (3 min) then 40 cycles of 98 °C (15 s), 58 °C (60 s), 72 °C (60 s). Standard curves (10^5^ to 10^8^ copies per µL) were prepared from plasmid DNA containing *nifH* and product specificity confirmed by endpoint melt curve analysis.

A three step PCR was used to prepare the *nifH* library^72^. *nifH* was amplified using IGK3/DVV in 10 µL of MyTaq Red Mix (Bioline), 0.8 µL of each primer (10 µM), 0.8 µL of DNA template and 7.6 µL MBGW on a T100 Cycler (Bio-Rad) at (1) 94 °C (5 min); (2) 36 cycles of 94 °C (30 s), 57 °C (45 s), 72 °C (30 s); (3) 72 °C (10 min). These PCR products were then re-amplified with IGK3/DVV appended with overhang MiSeq adaptors in 25 µL containing 12.5 µL of MyTaq Red Mix (Bioline), 1 µL of each primer (10 µM), 1 µL of amplicons from the first step as template and 9.5 µL of MBGW. The PCR programme was: (1) 94 °C (4 min); (2) 12 cycles of 94 °C (30 s), 57 °C (45 s), 72 °C (30 s); (3) 72 °C (7 min). PCR products were cleaned using AMPure XP beads and multiplexing barcodes added by the Index PCR in 25 µL containing 12.5 µL of MyTaq Red Mix (Bioline), 0.5 µL of each primer (10 µM), 0.5 µL of DNA template and 11 µL of MBQW at (1) 95 °C (3 min); (2) 8 cycles of 98 °C (20 s), 57 °C (15 s), 72 °C (15 s); (3) 72 °C (5 min). Final amplicons were quantified (Qubit 2.0 Fluorometer (Invitrogen)) and normalised to 4 nM (SequalPrep Normalization Plate Kit, Invitrogen), combined and sequenced (Illumina MiSeq, 300 base paired-ends).

### Sequence processing pipeline and phylogenetic analysis

Paired-end de-multiplexed files were imported into QIIME2 (v.2021.11) on the Apocrita HPC facility at Queen Mary University of London^73^ (Supplementary Fig. 9) and processed using DADA2 to trim primers, remove low-quality sequences and chimeras^74^. Sequences were clustered into species-level OTUs at 95% similarity^72^, singletons and sequences >356 bp or <333 bp and low-abundance OTUs (<20 reads and in 3 samples or less) were removed. Amino acid sequences were aligned to known *nifH* and non-*nifH* references and a phylogenetic tree constructed using COBALT^75^. The primers IGK3/DVV can amplify non-*nifH* homologues including the chlorophyll synthesis genes *BChL* and *ChlL* and these were identified after translating the OTU sequences using “Translate” in MEGA (version 10.2.2). Translation initiation site adjustment and frameshifts were detected using blastp^76^. Amino acid sequences were aligned to known *nifH* and non-*nifH* references and a phylogenetic tree constructed using COBALT^75^. The non-*nifH* OTUs were identified using distinct non-conservative short sequence motifs and visualised using the iTOL tool^77^ that appeared as two separate clusters on the phylogenetic tree (*see* Supplementary Fig. 10). Approximately 82% of the sequences were non-*nifH* homologues, which is common when using general *nifH*- primers^71^. Non-*nifH* sequences were removed and q-PCR estimates of *nifH* gene abundances were corrected for the proportion of non-*nifH* sequences in each sample.

### Statistical analysis

Statistical analysis and plotting were performed in R^78^ using RStudio (Version 1.3.1093). We used generalised additive mixed effects models (GAMMs)^79^ to characterise the seasonal patterns in N_2_ and N_2_O saturation, fitting sampling month as a fixed effect and each replicate pond as random effects. We included an interaction term for sampling month by gas (N_2_ or N_2_O) to explore any distinct seasonality in N_2_ and N_2_O saturation. Models were ranked by the small sample-size corrected Akaike Information Criterion (AICc) using the ‘MuMIn’ package (Supplementary Table 1).

Rate data for total ^15^N_2_O reduction and ^15^N assimilation were skewed, potentially due to normalising to unit dry biomass which may not account for the true abundance of N_2_ and N_2_O fixers. Therefore, we present 95% of data (2.5% to 97.5% percentiles) for both datasets (Figs. 2a, c, 3a and 4a) and fitted quantile regression models (‘quantreg’^80^) rather than mean regression models to the full dataset for rate of ^15^N assimilation to minimise any bias from outliers (median regression lines, Fig. 4a). The difference between the temperature response of ^15^N_2_ and ^15^N_2_O was compared using the ‘emmeans’ package.

*nifH* Shannon diversity was calculated using the ‘estimate_richness’ function in the ‘phyloseq’ package^81^ and any changes in the *nifH* community calculated using the ‘adonis’ function from the ‘Vegan’ package^82^ (with Original UniFrac distance). Principal Coordinates Analysis (PCoA) was used to test the significance of either incubation day or excess N_2_O on *nifH* community composition by Permutational multivariate analysis of variance (PERMANOVA) and redundancy analysis (RDA) to ordinate N_2_O reduction and *nifH* relative abundance.

### Reporting summary

Further information on research design is available in the Nature Portfolio Reporting Summary linked to this article.

### Supplementary information

Supplementary Information

Peer Review File

Reporting Summary

### Source data

Source Data

## Data Availability

Data generated in this study are provided in the Source Data file. Source data are provided with this paper. The DNA sequences are in the National Center for Biotechnology Information database, under BioProject ID PRJNA984972. Source data are provided with this paper.
