# Supplementary material for: Direct biological fixation provides a freshwater sink for N2O
Source: Nat Commun. 2023 Oct 25;14:6775. doi: 10.1038/s41467-023-42481-2 (PMC10600110; doi:10.1038/s41467-023-42481-2)
Supplement: Supplementary file 1 — Supplementary Information [file 41467_2023_42481_MOESM1_ESM.pdf]

Supplementary information

Direct biological fixation provides a freshwater sink for N_2O

Authors: Yueyue Si[1], Yizhu Zhu[1], Ian Sanders[1], Dorothee Beate Kinkel[1], Kevin J Purdy[2], Mark Trimmer[1]*

Affiliations:

[1]School of Biological and Behavioural Sciences, Queen Mary, University of London, London E1 4NS, UK.

[2]School of Life Sciences, University of Warwick, Coventry CV4 7AL, UK.

*Correspondence to: Mark Trimmer m.trimmer@qmul.ac.uk

Supplementary Text 1 | Gibbs free energy changes for the equations discussed in the main text

First, we present free energy changes for N_2O in relation to canonical N_2 fixation and NO_3^- assimilation under standard conditions (ΔG° at 298.15 K) and then for freshwater at 10 °C (ΔG at 283.15 K), see details below:

(Equation S1) $0.5N_2O+1.5H_2O \rightarrow 1NH_3 + 1O_2$ $\Delta G° = +277$ kJ per mol NH_3

$$\Delta G = \Delta G° + RT\ln\frac{[NH_3]^1[O_2]1}{[N_2]^{0.5}[H_2O]^{1.5}} = +247 \text{ kJ per mol } NH_3$$

(Equation S2) $0.5N_2 + 1.5H_2O \rightarrow 1NH_3 + 0.75O_2$ $\Delta G° = +329$ kJ per mol NH_3

$$\Delta G = \Delta G° + RT\ln\frac{[NH_3]^1[O_2]^{0.75}}{[N_2]^{0.5}[H_2O]^{1.5}} = +291 \text{ kJ per mol } NH_3$$

(Equation S3) $1NO_3^-+3H^++2e^-\rightarrow 1NH_3+1.5O_2$ $\Delta G° = +85$ kJ per mol NH_3

$$\Delta G = \Delta G° + RT\ln\frac{[NH_3]^1[O_2]1.5}{[NO_3^-]^1[H^+]^3[e^-]^2} = +241 \text{ kJ per mol } NH_3$$

Here, ΔG^0 is the standard Gibbs free energy, at 298.15 K, 1 bar, with 1 M of each reactant and product and is calculated as the sum of the standard Gibbs free energies of formation of each component ($\Delta_f G^0$), where n is the number of molecules:

(Equation S4) $$\Delta G^0 = \sum n\Delta_f G^0(\text{products}) - \sum n\Delta_f G^0(\text{reactants})$$

Then, ΔG is the Gibbs free energy for temperate freshwater at pH 7 and 10 °C (283.15 K) calculated under our defined conditions according to the Nernst equation as:

(Equation S5) $$\Delta G = \Delta G° + RT\ln\frac{[\text{product}]^n}{[\text{reactant}]^n}$$

Where R is the gas constant (0.00831 kJ mol^{-1} K^{-1}), T is temperature, defined as 283.15 K, n is the number of molecules, [product] and [reactant] are concentrations of products and reactants, respectively. For the gas components in reactions S1 to S3 we used concentrations at 100% atmospheric equilibration for freshwater: $[O_2]$ = 346 µM, $[N_2]$ = 630 µM and $[N_2O]$ = 13 nM. We defined $[NH_3]$ and $[NO_3^-]$ to be 1 µM and 10 µM, respectively. As pH = 7, $[H^+]$ and $[e^-]$ were both 10^{-7} M and $[H_2O]$ = 1 regardless of any defined condition. Note that the positive free-energy change (ΔG>0) in S1 to S3 demonstrates that fixing either N_2 or N_2O or assimilating NO_3^- requires external energy.

Next, we present the free energy changes for both the complete oxidation of CH_4 to CO_2 (S6) and then for 50% (ref[1]) of the C in CH_4 being assimilated into biomass (S7) again for both standard conditions and those typical of freshwater at 10°C:

(Equation S6) $1CH_4 + 2O_2 \rightarrow 1CO_2 +2H_2O$ $\Delta G° = -818$ kJ per mol CH_4

$$\Delta G = \Delta G° + RT\ln\frac{[CO_2]^1[H_2O]^2}{[CH_4]^1[O_2]^2} = -763 \text{ kJ per mol } CH_4$$

(Equation S7)
$1CH_4+1.5O_2 \rightarrow 0.5CO_2+0.5HCHO+1.5H_2O$

$\Delta G° = -559$ kJ per mol CH_4

$$\Delta G = \Delta G° + RT\ln\frac{[CO_2]^{0.5}[HCHO]^{0.5}[H_2O]^{1.5}}{[CH_4]^1[O_2]^{1.5}} = -522 \text{ kJ per mol } CH_4$$

Here we used $[O_2]$ = 346 1 μM, $[CO_2]$ = 1.6 mM, $[CH_4]$ = 1 μM and [HCHO] = 1 μM. In contrast to S1 to S3, the free-energy changes in S6 and S7 are negative ($\Delta G<0$) and oxidising CH_4 liberates energy. As per the main text, some 32% of the full energy yield from S6 is required to fix C from CH_4 into biomass (S7), with 56% of the remainder being required to fix N_2 (S2/S7) compared to 47% to fix N_2O (S1/S7).

Supplementary Text 2 | Estimating N_2 and N_2O fluxes, and *in situ* N_2O reduction in the ponds.

Estimating N_2 and N_2O fluxes across the water to air interface in the ponds

The wind speed around the ponds was mostly less than 3 m s^{-1} throughout the year (median 1.8 m s^{-1}), as recorded using a Datahog 2 logger connected to an on-site weather station (Skye Instruments). The gas transfer velocity is typically independent of such low wind speeds[2] and would possibly be underestimated if it were calculated based on such empirical gas exchange-wind speed relationships[2,3]. Here, we estimated the gas transfer velocity for N_2O (k_{N2O}, cm h^{-1}) using previously determined gas transfer velocities for CH_4 (k_{CH4}, cm h^{-1}) in the ponds[4] and the ratio of their respective Schmidt numbers (unitless):

(Equation S8) $$k_{N2O} = k_{CH4} \times \left(\frac{Sc_{N2O}}{Sc_{CH4}}\right)^{-0.66}$$

Where Sc_{N2O} and Sc_{CH4} are the Schmidt numbers for N_2O and CH_4 at *in situ* water temperature, respectively, with the corresponding exponent (-0.66) applied for the smooth water surface of the ponds[3]. N_2O flux across the water to air interface in the ponds (F_{N2O}, µmol m^{-2} d^{-1}) was then derived by:

(Equation S9) $$F_{N2O} = k_{N2O} \times (Cw_{N2O} - Ca_{N2O})$$

Where Cw_{N2O} and Ca_{N2O} are the measured and air-equilibrated concentrations of N_2O in the ponds, respectively. N_2 fluxes were derived in the same way as for N_2O using the appropriate Schmidt and equilibration numbers.

As a result, N_2O flux into our ponds was -1.33 µmol N_2O m^{-2} d^{-1}, on average, with a range of -3.65 to 0.02 µmol N_2O m^{-2} d^{-1}, including low emissions to the atmosphere in summer. While N_2 flux into the ponds was -3,934 µmol N_2 m^{-2} d^{-1}, on average.

Estimating *in situ* rates of N_2O reduction in the ponds required to balance the N_2O flux

We characterised the kinetic effect of N_2O concentration on the rate of N_2O reduction experimentally using incubations with floating biomass enriched with different concentrations of N_2O from 9.2 nM to 20,000 nM (Supplementary Fig. 4). We then estimated rates of N_2O reduction (y, nmol g^{-1} DW h^{-1}) at *in situ* concentrations of N_2O in the ponds (x, nM) according to:

(Equation S10) $$\ln(y) = 0.95 \times \ln(x) - 4.96$$

Therefore, with an ambient concentration of N_2O of 10 nM at the annual average temperature of 15°C in the pond water (Supplementary Fig. 2), N_2O reduction would be 0.06 nmol g^{-1} DW h^{-1}, on average, where DW denotes the dry weight of floating biomass. Although

we measured the conversion factor of dry to wet weight of the biomass collected from the ponds, as floating biomass is non-homogenous both within a pond and between different ponds, e.g., percentage volume infested (PVI, %) of the filamentous algae ranged from 0.1 to 40 in the ponds, estimating N_2O reduction in the ponds with the weight of floating biomass would be difficult. As benthic biomass is distributed relatively-evenly in the ponds, and from our incubations, rates of total $^{15}N_2O$ reduction were consistent between the floating and benthic biomass ($p = 0.9$, t-test), instead we applied the kinetic function (1) to the benthic biomass.

First, based on the conversion factor of wet weight to dry weight (WW/DW = 12.17), N_2O reduction per unit wet weight would be 0.06 nmol g^{-1} DW h^{-1}/12.17 = 0.005 nmol g^{-1} WW h^{-1}. Then, based on the estimated wet-bulk density of benthic biomass (~ 1.01 g cm^{-3})[5], we converted the N_2O reduction rate to per unit volume of biomass as: 0.005 nmol g^{-1} WW h^{-1} × 1.01 g cm^{-3} = 0.005 nmol cm^{-3} h^{-1}. Finally, using the depth of oxic benthic biomass (~ 0.006 m)[4] in the ponds, we estimated *in situ* rates of N_2O reduction in the ponds per unit area of biomass as: 0.005 nmol cm^{-3} h^{-1} × 1,000,000 × 0.006 m = 31.12 nmol m^{-2} h^{-1}, which is equivalent to 0.75 μmol m^{-2} d^{-1}.

Supplementary Figure 1 | The measured concentration of dissolved N_2O in the pond water and equilibration concentration of N_2O as a function of temperature. We fitted a simple first-order linear model to the measured N_2O concentrations (blue line, slope = -0.36, $p < 0.001$, $n = 230$ samples) which we can see differs to the equilibration N_2O concentrations (black line, 2nd order quadratic) and in the shape of their response to temperature. Although we measured the temperature in each pond at the time of each gas sampling and are confident of little variation, we have applied a ± 1 °C variation in temperature to illustrate possible uncertainties in the equilibration concentration (shaded grey area). The measured and equilibration concentrations are more different at colder temperatures, showing that N_2O is more undersaturated in the cold.

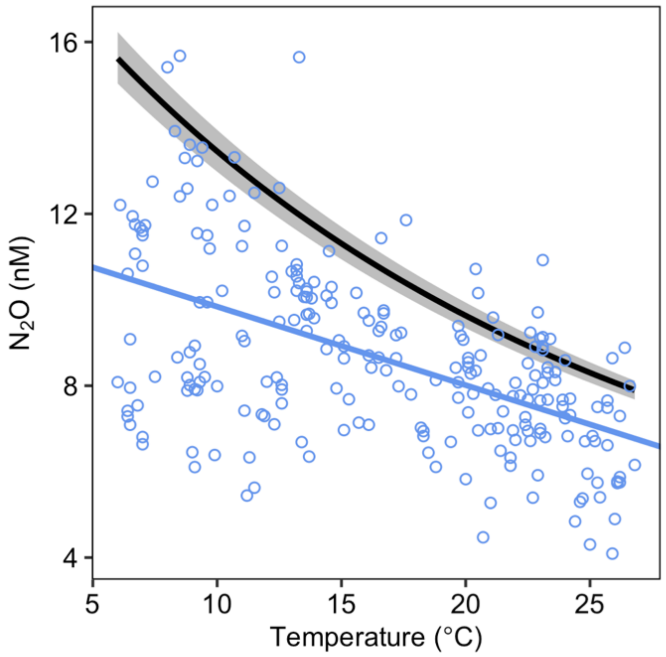

Supplementary Figure 2 | Correlation between saturation of dissolved O_2 and N_2, N_2O, or temperature in the pond water. a, Correlation between dissolved oxygen saturation and N_2O saturation in the ponds. **b**, Correlation between dissolved oxygen and N_2 saturation in the ponds. **c**, Correlation between dissolved oxygen saturation and temperature in the ponds indicating maximum primary production in spring and summer. The lines in **b** and **c** are simple first order linear regression models. n = 175, n = 156, and n = 180 samples in **a**, **b**, and **c**, respectively (n represents data for 9 months in 20 ponds).

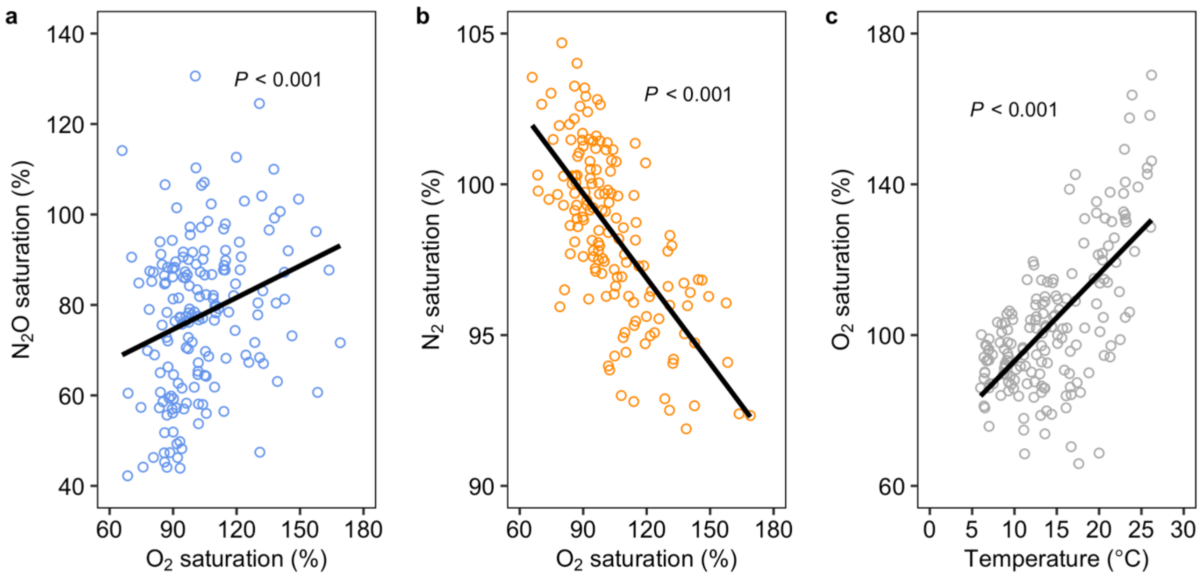

Supplementary Figure 3 | View of the experimental ponds and biomass types used in the incubations. **a**, The artificial, experimental ponds in East Stoke, Dorset, UK, established in September 2005 (ref[6]). **b**, Close-up showing floating assemblages, dominated by *Oedogonium* sp., on the surface of the ponds. **c**, Close-up showing organic benthic biomass covering the bottom of a pond. **d**, Microscopy image showing filaments of *Anabaena sp*. – a N_2-fixing cyanobacterium, identified in the floating assemblages in the ponds. Copyright: **a** – **c,** Yueyue Si, **d**, Danielle Marchant.

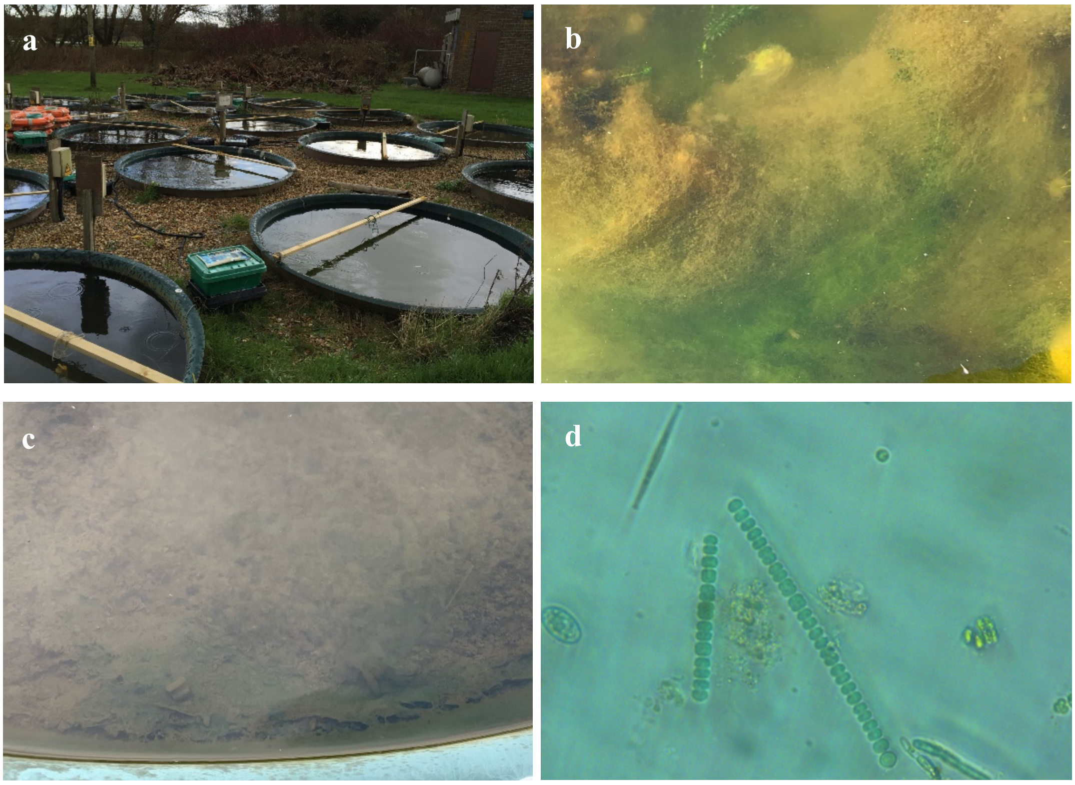

Supplementary Figure 4 | Seasonal changes in daily average temperature in the pond water combined for the warmed and ambient ponds between January 2019 to December 2021. The annual average temperature in the ponds was 15.4°C. *n* = 6546 measurements (18 ponds, as the temperature loggers failed in 2 out of the 20 ponds).

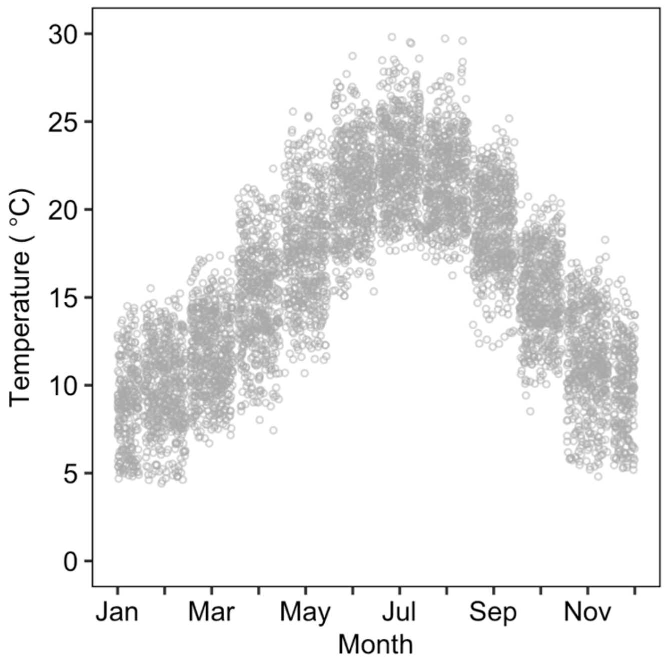

Supplementary Figure 5 | The ratio of $^{30}N_2$ to total N_2 expressed as raw signal of *m/z* 30 to the total *m/z* (Σ28+29+30) in all of our biomass incubations. a, floating biomass and **b,** benthic biomass. Overall, the ratio of $^{30}N_2$ to total N_2 from $^{15}N_2O$-amended incubations increased with benthic biomass but no significant enrichment was found with floating biomass. We used a mixed-effect model to characterise the overall ratio of $^{30}N_2$ to total N_2 in floating and benthic incubations accounting for variation between different batches of measurement by including each batch as a random effect on the intercept. On average, the production of $^{30}N_2$ from $^{15}N_2O$ in the benthic incubations was 0.27 µM (Fig. 3b, below), which would result in 0.29 nmol N g^{-1} d^{-1} of ^{15}N assimilation if all of that $^{30}N_2$ was subsequently fixed. In contrast, this 0.29 nmol N is far lower than the rate of ^{15}N assimilation that we measured (5.3 nmol N g^{-1} d^{-1}, Table 1, main text) with $^{15}N_2O$, which means that even in the benthic biomass incubations that showed significant $^{15}N_2$ production, $^{15}N_2O$ assimilation still appears to have been mainly direct. $n = 194$ and $n = 193$ incubations in Control and $^{15}N_2O$-amended incubations in **a**, $n = 271$ and $n = 268$ incubations in Control and $^{15}N_2O$-amended incubations in **b**, respectively. The horizontal line inside each box is the mean and shaded area the 95% C.I..

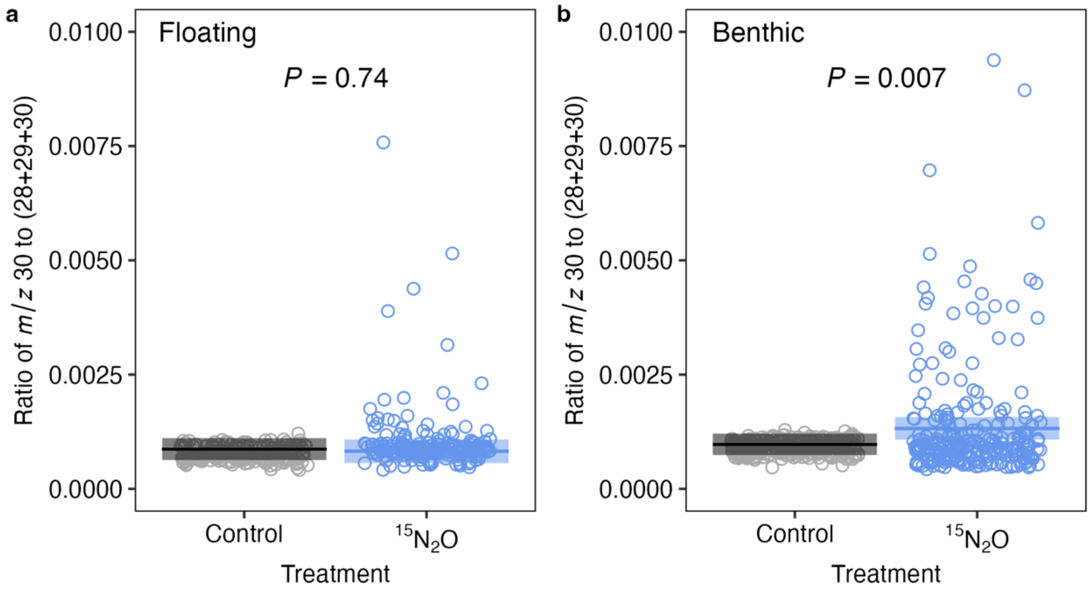

Supplementary Figure 6 | Kinetic effect of N_2O concentration on the rate of N_2O reduction. The rate of N_2O reduction increased as a function of N_2O concentration (9.2 nM – 20,000 nM) in incubations with floating biomass (*n* = 81 incubations, floating biomass collected from 12 ponds). We used mixed-effect models to characterise the overall kinetic effect of N_2O concentration on the rate of N_2O reduction and we accounted for variation across ponds by including each pond as a random effect on the intercept. The significance of slope was tested using log-likelihood-ratio comparing full to simpler, reduced models (***: $p<0.001$).

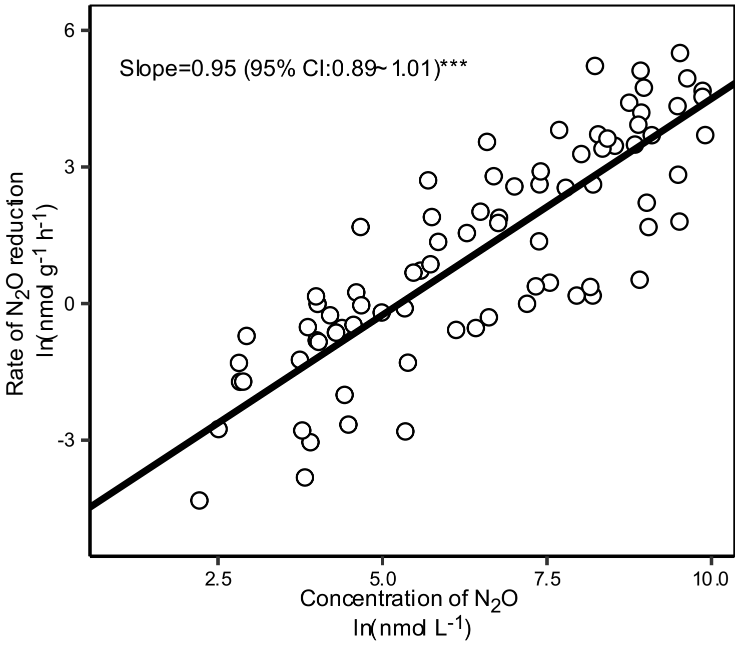

Supplementary Figure 7 | Dissolved inorganic nitrogen in the 25-day laboratory incubation. a, In the first three days of the incubation the concentration of nitrate and nitrite increased 1.5-fold more rapidly in the pond water enriched with N_2O than in the controls (0.6 µM vs 0.4 µM, N_2O enriched vs controls, mean to mean respectively). **b**, The concentration of ammonium increased throughout to a plateau at ~0.3 µM. The solid dots represent the mean concentration of nitrate and nitrite (panel **a**) or ammonium (panel **b**) with standard error bars (n = 80 incubations, biomass from 10 experimental ponds was enriched with N_2O and biomass from the other 10 ponds left unamended as controls). Replicates were sacrificed at 0, 3, 10 and 25 days of incubation. Note, incubations with biomass from one pond had concentrations of nitrate and nitrite (> 4 µM) and ammonium (> 3 µM) far greater than the other nine ponds (both N_2O enriched and controls) probably due to contamination in the serum bottle and were excluded from the data presented here. Data plotted in **a** and **b** are means ± s.e.

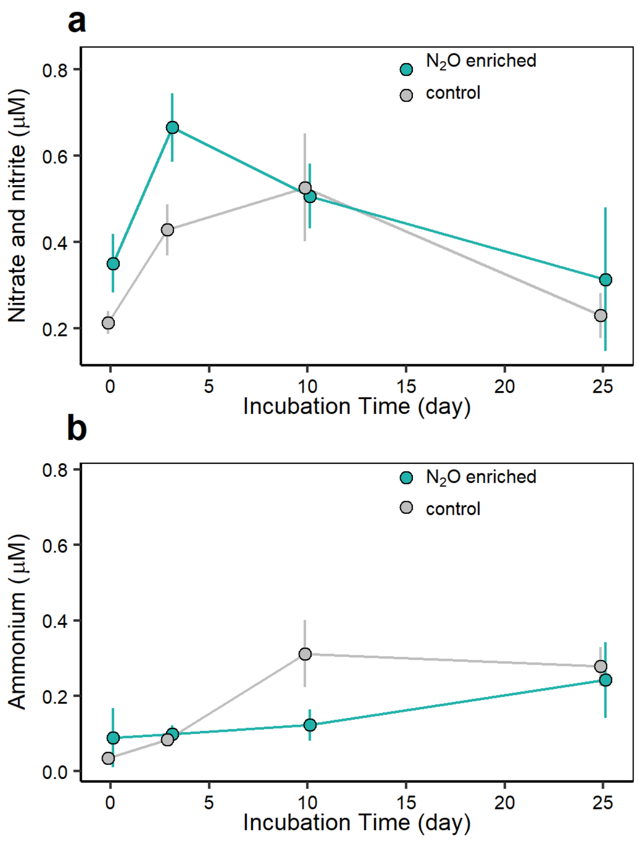

Supplementary Figure 8 | Oxygen production in the 25-day laboratory incubation. a, Typical average patterns of oxygen production over time with floating biomass after two days of incubation either with or without N_2O enrichment (*n* = 10 incubations, dissolved oxygen measured at ~2-hour intervals). **b**, Daily maximum oxygen concentration started to drop on the 25th day of incubation indicating a decline in photosynthetic vigour of biomass (*n* = 1948 measurements, dissolved oxygen in the 20 serum bottles measured at ~2-hour intervals every day for 25 days). Data plotted in **b** are means ± s.e.

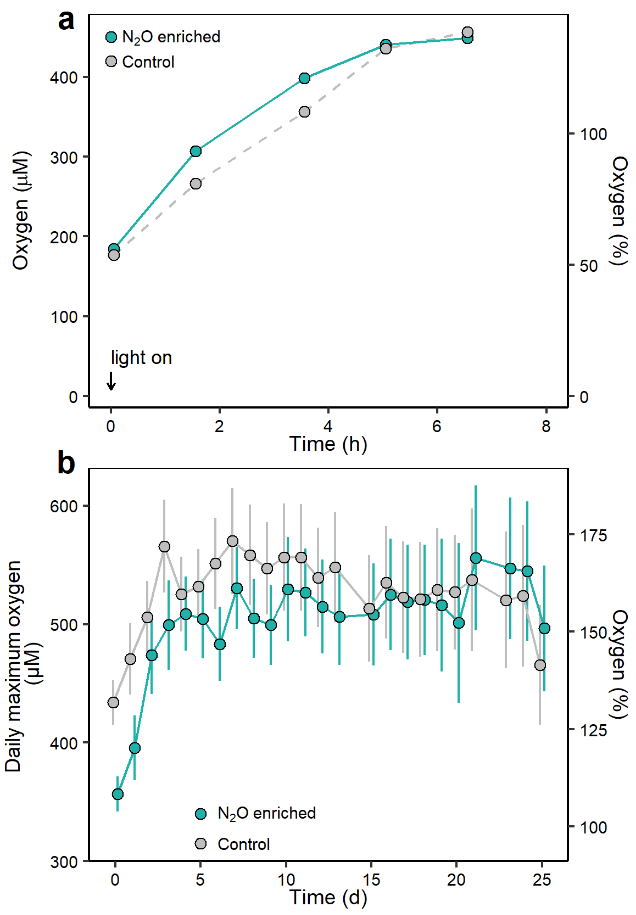

Supplementary Figure 9 | As all 40 samples (biomass collected from ten experimental ponds sacrificed after 0, 3, 10 and 25 days of incubation) were capable of reducing N_2O (*see* Fig. 5a in the main text), the *nifH* OTUs related to N_2O reduction would likely be well-represented across the N_2O-enriched samples. **a**, (next page) the *nifH* community was analysed following the steps in the flow chart. After removing OTUs of the wrong size or present as singletons, only 894 out of the 1,840 OTUs were regarded as well-represented, being represented by > 20 reads and in at least three samples. Therefore, only these well-represented 894 were used in the downstream analysis while the other 946 OTUs were discarded (box labeled by †). **b**, The 946 OTUs discarded from further analysis were uncommon across the total of 80 samples (40 enriched with N_2O and their 40 un-enriched controls). In fact, 37% of the OTUs discarded were present in only two samples and more than 80% of the OTUs discarded were present in at most five samples, 17% were present in six to ten samples and only 2% were in more than 15 samples. However, none of discarded OTUs were present in more than 20 samples. **c**, The 5 candidate OTUs for N_2O fixation (in the black box), out of the total 72 OTUs ordinated to the initial rates of N_2O reduction (Fig.5b of the main text), were not only common in the ten samples used to determine the initial rates of N_2O reduction (s1 to s10) but also in all 40 samples enriched with N_2O. The three Cyanobacterial OTUs, i.e., *Pegethrix*-like OTU392, OTU394 and *Fischerella*-like OTU396 were present in more than 65% of the samples. The two *Methylomonas*-like OTU444 and OTU462 were less common but still present in more than 30% of the samples. The numbers in brackets give the percentage of samples that each OTU was present in.

a

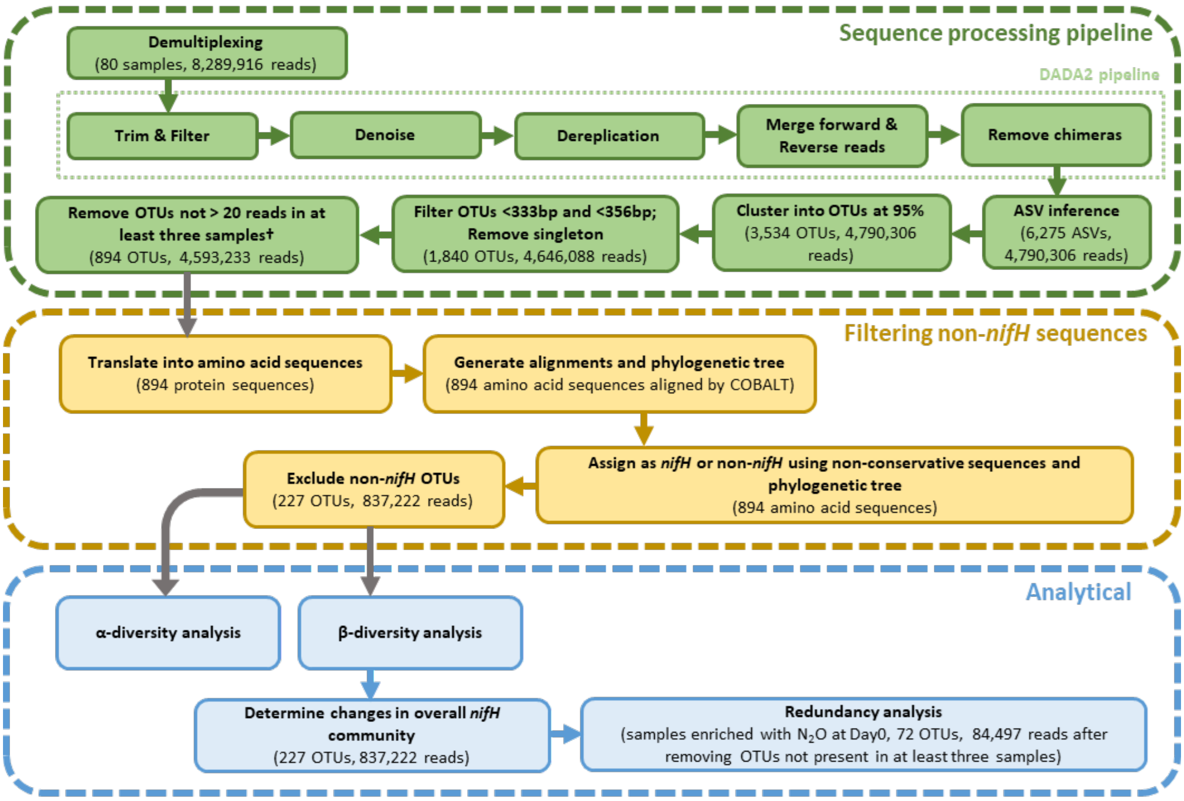

† We defined well-represented OTUs as OTUs represented by more than 20 reads in at least three samples and only well-represented OTUs were used in downstream analysis.

b

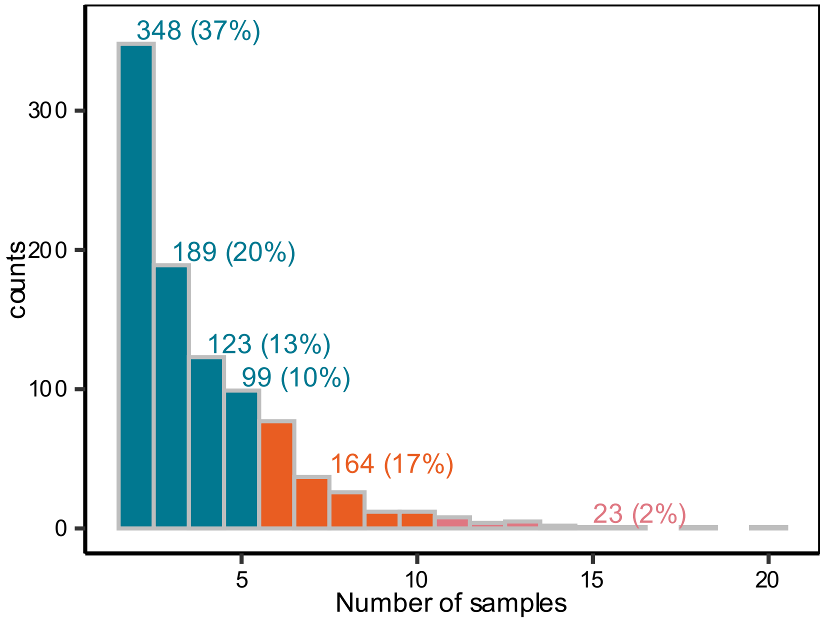

c

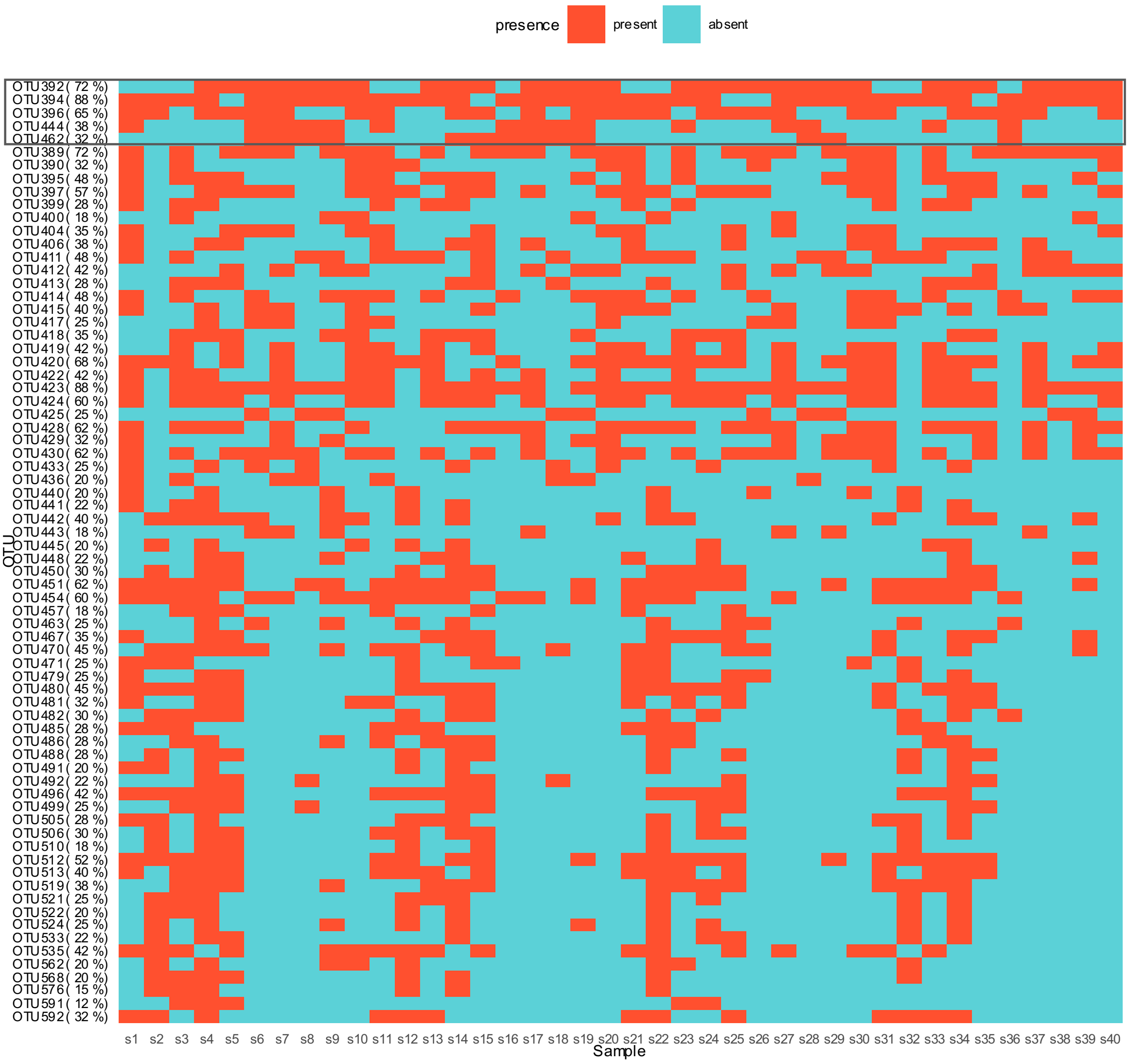
presence
present
absent
OTU392(72 %)
OTU394(88 %)
OTU396(65 %)
OTU444(38 %)
OTU462(32 %)
OTU389(72 %)
OTU390(32 %)
OTU395(48 %)
OTU397(57 %)
OTU399(28 %)
OTU400(18 %)
OTU404(35 %)
OTU406(38 %)
OTU411(48 %)
OTU412(42 %)
OTU413(28 %)
OTU414(48 %)
OTU415(40 %)
OTU417(25 %)
OTU418(35 %)
OTU419(42 %)
OTU420(68 %)
OTU422(42 %)
OTU423(88 %)
OTU424(60 %)
OTU425(25 %)
OTU428(62 %)
OTU429(32 %)
OTU430(62 %)
OTU433(25 %)
OTU436(20 %)
OTU440(20 %)
OTU441(22 %)
OTU442(40 %)
OTU443(18 %)
OTU445(20 %)
OTU448(22 %)
OTU450(30 %)
OTU451(62 %)
OTU454(60 %)
OTU457(18 %)
OTU463(25 %)
OTU467(35 %)
OTU470(45 %)
OTU471(25 %)
OTU479(25 %)
OTU480(45 %)
OTU481(32 %)
OTU482(30 %)
OTU485(28 %)
OTU486(28 %)
OTU488(28 %)
OTU491(20 %)
OTU492(22 %)
OTU496(42 %)
OTU499(25 %)
OTU505(28 %)
OTU506(30 %)
OTU510(18 %)
OTU512(52 %)
OTU513(40 %)
OTU519(38 %)
OTU521(25 %)
OTU522(20 %)
OTU524(25 %)
OTU533(22 %)
OTU535(42 %)
OTU562(20 %)
OTU568(20 %)
OTU576(15 %)
OTU591(12 %)
OTU592(32 %)
OTU
s1 s2 s3 s4 s5 s6 s7 s8 s9 s10 s11 s12 s13 s14 s15 s16 s17 s18 s19 s20 s21 s22 s23 s24 s25 s26 s27 s28 s29 s30 s31 s32 s33 s34 s35 s36 s37 s38 s39 s40
Sample

Supplementary Figure 10 | In total, 894 well-represented OTUs were amplified using the IGK3/DVV primers. The non-*nifH* OTUs were characterised by visual inspection of amino acid sequence alignments with known NifH or non-NifH sequences. **a,** for example (next page), alignment of amino acid sequences for 19 out of the 894 OTUs demonstrated clear separation between NifH (green box) and non-NifH (black box) in their non-conservative regions (red arrows). **b,** Final phylogenetic tree of the 894 OTUs demonstrating that the 667 non-*nifH* OTUs formed two distinct clusters (shaded in grey). The phylogenetic tree was derived from amino acid sequences using the Maximum Likelihood method.

a

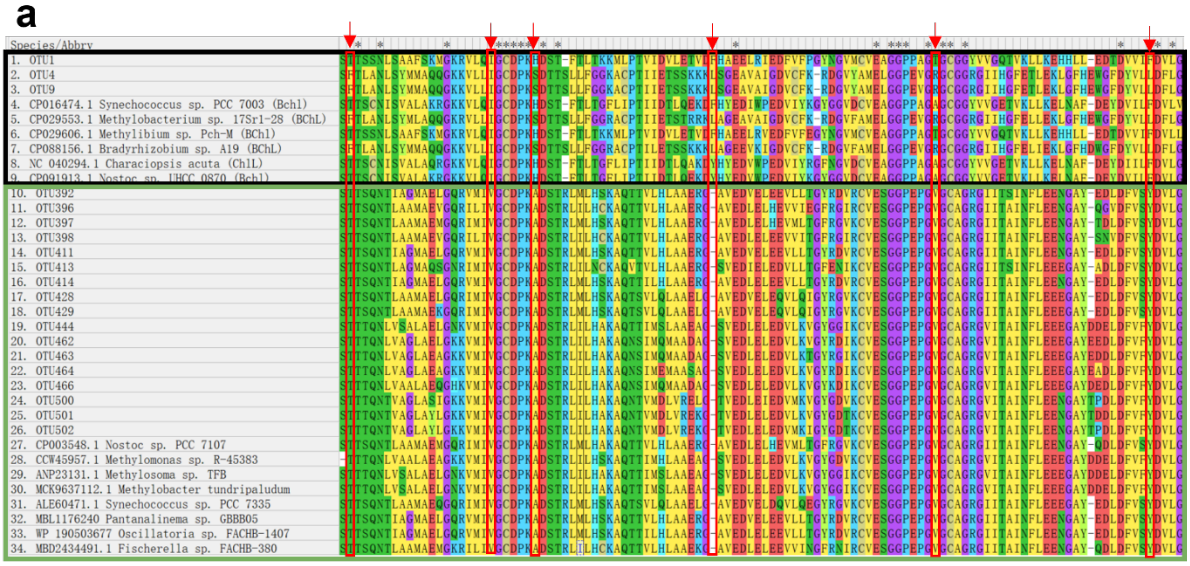

b

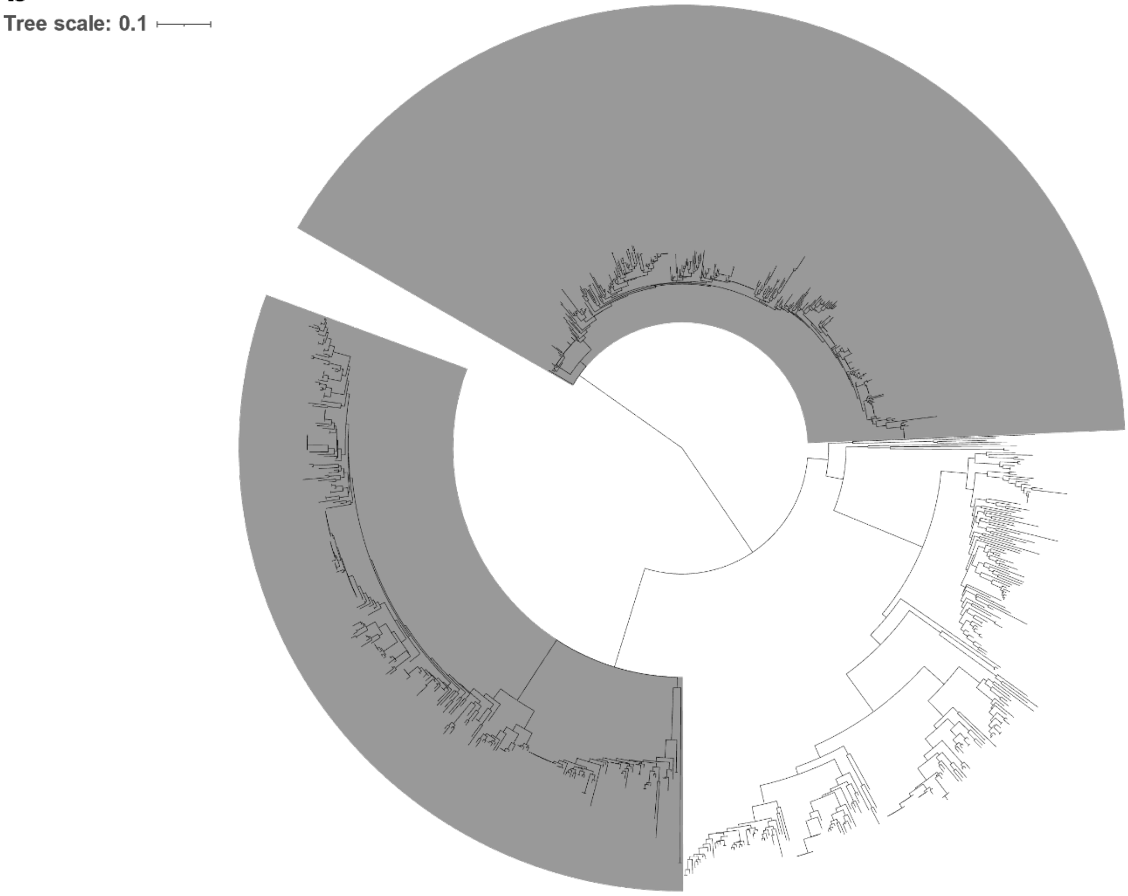

Supplementary Figure 11 | The *nifH* community at four time points during the 25-day incubation. There were no detectable changes in the overall *nifH* community (227 OTUs) across samples incubated with either excess N_2O or in the controls either at 3, 10 or 25 days (**a**, n = 40) or, individually, after 3 (**b**, n = 20), 10 (**c**, n = 20) or 25 (**d**, n = 20) days, respectively. The PCoA ordination was obtained using the Original UniFrac distance and the numbers in brackets represent the proportion of variance explained by the first (PCoA1) or second axis (PCoA2), respectively. The p values were calculated using PERMANOVA and the adonis function (see Methods).

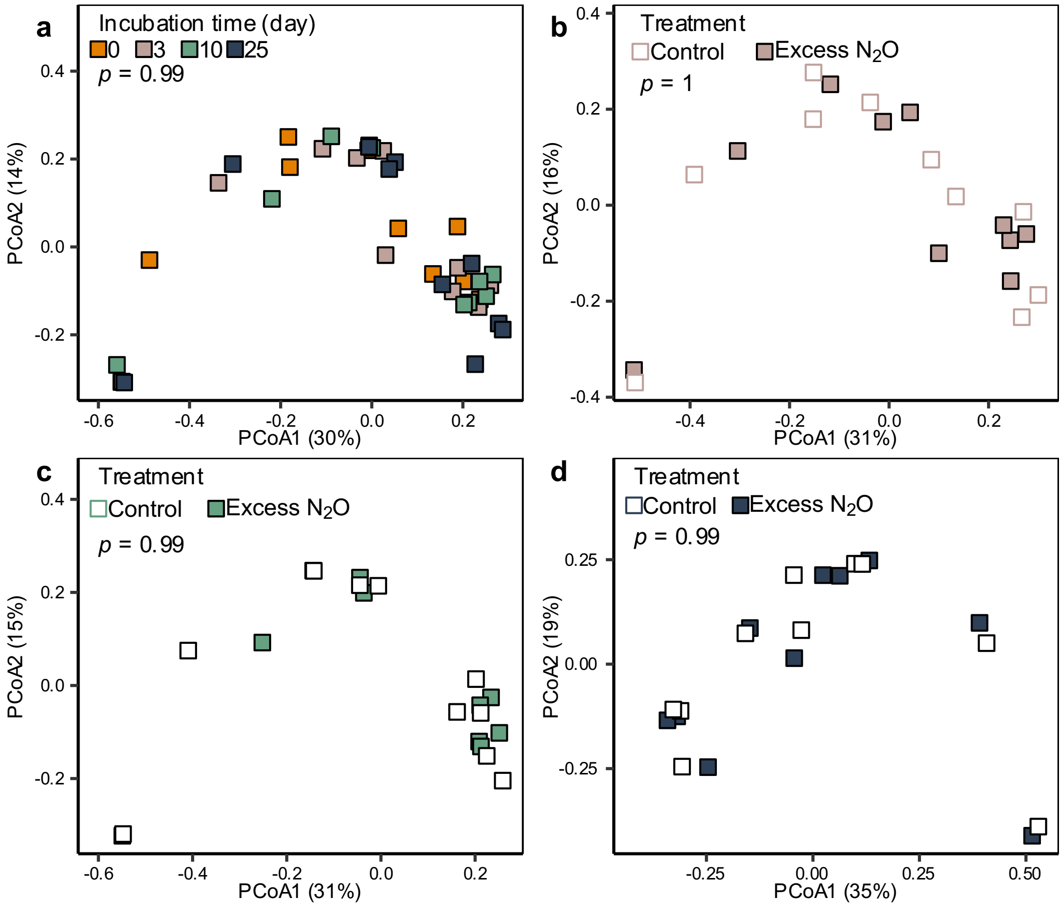

Supplementary Figure 12 | Meta-analysis fitting published rates of biological N_2 fixation as a function of temperature in incubations with biomass from both aquatic[7-10] and terrestrial[11-16] communities. Biological N_2 fixation clearly increases at higher temperatures with a consistent temperature response for both aquatic and terrestrial communities. We visualized the data using the "Visreg" package in R[17] showing the best fitting linear mixed-effect model (blue line, Supplementary Table 2) and partial residuals (grey circles). The rate of biological N_2 fixation was natural log (ln) transformed. Data were excluded from the analysis where the rate of N_2 fixation reached a plateau or had started to decline at the highest temperatures (e.g., typically >25°C to 30°C)[7,9,12,14]. As the studies used different normalization units: e.g., µmol C_2H_4 (mg Chl a)$^{-1}$ h^{-1} for ref. 1; µmol N d^{-1} L^{-1} for ref. 4; and µmol C_2H_4 h^{-1} (g dry wt)$^{-1}$ for ref. 7 and 11, the variation in unit was accounted for by including each study as a random effect on the slope and the intercept (Supplementary Table 2). *n* = 151 data points from 10 studies[7-16].

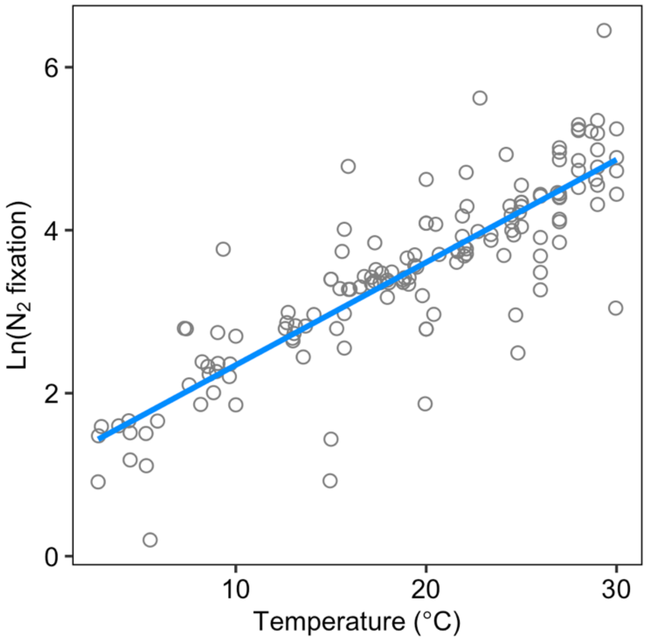

Supplementary Figure 13 | N_2O measured on our Thermo Delta V Plus CF-IRMS showing that the increase in total peak area to nmol $^{15}N_2O$ (total peak area dominated by *m/z* 46) or nmol $^{14}N_2O$ (total peak area dominated by *m/z* 44) is the same (p = 0.88, n = 17 and n = 5 in $^{14}N_2O$ and $^{15}N_2O$ calibrations, respectively). All vials were pre-flushed with air which resulted in an intercept of 0.38. The lines are simple first order linear regression models with 95% C.I. on the slopes (sensitivity) in brackets.

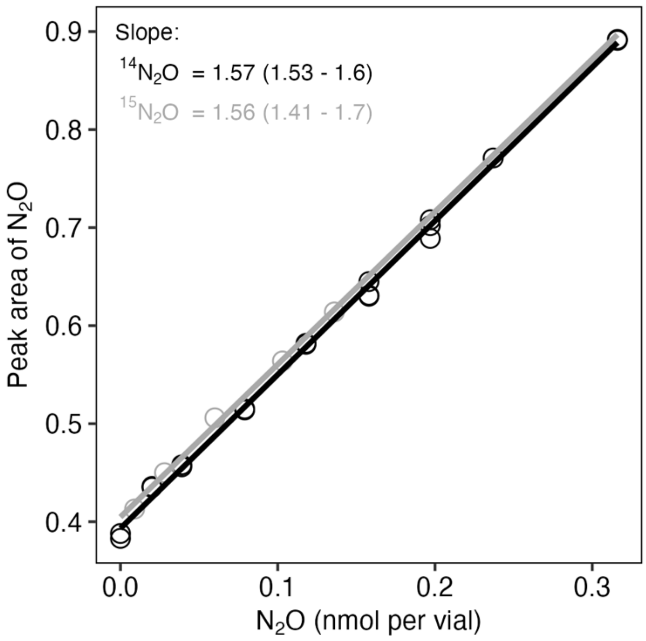

Supplementary Figure 14 | Interference from formaldehyde in the sulfamic-acid assay for converting $^{15}NO_3^-$ to $^{15}N_2$ (a two-step process comprising the initial conversion of $^{15}NO_3^-$ to $^{15}NO_2^-$ (ref [18]) followed by the conversion of $^{15}NO_2^-$ to $^{15}N_2$ (ref[19])). Over a concentration range of $^{15}NO_3^-$ from 0 to 1.5 µM, 50% (w/v) formaldehyde decreased the conversion of $^{15}NO_3^-$ to $^{15}N_2$ by 48% ($p < 0.001$, $n = 6$). As the interference from formaldehyde was consistent over the tested concentration range of $^{15}NO_3^-$, we prepared calibration curves by adding the same amount of formaldehyde to standards as the incubations to calibrate the concentrations of $^{15}NO_3^-$. The lines are simple first order linear regression models with 95% C.I. in brackets.

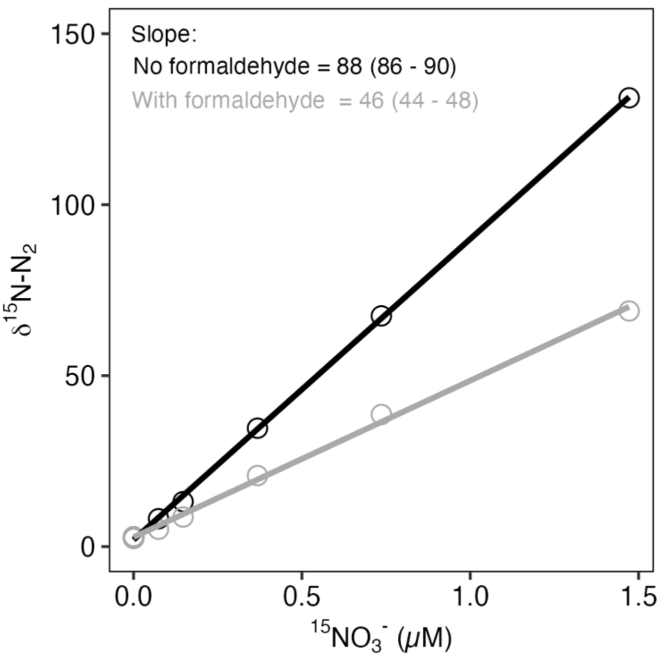

Supplementary Table 1 | Multi-GAMM selection for exploring seasonal patterns in N_2O and N_2 saturation over the year (main text, Fig. 1, a-c). Here, we treated both Month (i.e., seasonal pattern) and either gas (N_2 or N_2O) as fixed effects and fitted different smooth terms s() i.e., shape or pattern, along with a random intercept (1|Pond) to account for variation among the 20 experimental ponds. The term 'by=Gas' within s() denotes a different shape for the smooth term describing saturation for either N_2 or N_2O, whereas '+Gas' denotes a different intercept (i.e., median saturation) for either gas. Models were ranked by the corrected Akaike Information Criterion (AICc) and the best fitting models (in **bold**) to the data were judged as those with the lowest AICc. Here the best fitting model (**M0**) showed that N_2 and N_2O had different seasonal saturation patterns, with different median saturation values. We then further validated whether the seasonal patterns for N_2 or N_2O saturation were significant by modelling the data separately for N_2 and N_2O. Models M5 and M7 were compared to their respective null models (M6 and M8, which only have an intercept denoted by 1) to evaluate whether including the smooth term 'Month' improved model fit. The lower AICc in **M5** and **M7**, compared to their null models, suggested that both N_2 and N_2O saturation showed strong seasonality. n = 230 and n = 215 for N_2O and N_2, respectively (11 months for 20 ponds, with N_2O data for September measured in two different years). Models were compared to the best model in each panel using the Log-likelihood ratio test (LogLik, d.f., degrees of freedom) showing Chi-squared statistic (χ^2) and the corresponding p-value (p, two-sided).

Model	d.f.	AICc	LogLik	χ^2	p
**N_2 or N_2O Saturation**					
**M0: Sat~s(Month, by=Gas)+Gas**	**8**	**3226.9**	**-1605.3**		
M1: Sat~s(Month)+Gas	6	3389.4	-1688.6	166.63	<0.001
M2: Sat~Gas	4	3480.0	-1735.9	261.28	<0.001
M3: Sat~s(Month, by=Gas)	7	3555.7	-1770.7	330.83	<0.001
M4: Sat~s(Month)	5	3636.9	-1813.4	416.16	<0.001
**N_2O saturation**					
**M5: N_2O.sat1~s(Month)**	**5**	**1824.8**	**-906.9**		
M6: N_2O.sat2~1	6	1946.8	-970.3	126.67	<0.001
**N_2 saturation**					
**M7: N_2.sat1~s(Month)**	**5**	**983.3**	**-486.5**		
M8: N_2.sat2~1	6	1019.6	-506.7	40.45	<0.001

Supplementary Table 2 | Sampling dates (year & month) for saturations of N_2O and N_2 in the ponds.

Year	Month	Gas measured
2019	11	N_2O
2020	8, 9, 12	N_2O, N_2
2021	6, 7, 9, 10	N_2O, N_2
2022	1, 2, 3, 4	N_2O, N_2

Supplementary Table 3 | Meta-analysis for published rates of biological N_2 fixation (BNF) as a function of incubation temperature for biomass from both terrestrial and aquatic ecosystems (Supplementary Fig 10). Linear mixed-effect model selection included temperature and the interaction between temperature and ecosystem type as fixed effects, with a random intercept and slope (1+Temp|Study) to account for variation across the different studies[7-16]. Here, the best fitting model (**M0**) showed that the rate of N_2 fixation increased at higher temperatures, and that the temperature sensitivity for N_2 fixation was not different between aquatic and terrestrial ecosystems. Models were ranked by the Akaike Information Criterion (AIC) with the better models (in **bold**) having lower AIC values. M3 is the null model which only included an intercept, denoted by 1. lnBNF is the natural log (ln) transformed rate of biological N_2 fixation, Temp is temperature and Ecosystem is ecosystem type (terrestrial or aquatic). Models were compared to the best model in each panel using the Log-likelihood ratio test (LogLik, d.f. degrees of freedom) showing χ^2 (Chi-squared statistic) and the corresponding *p*-value (*p*, two-sided).

**Model**	**d.f.**	**AIC**	**LogLik**	χ^2	***p***
**M0: lnBNF~Temp**	6	346.3	-166.8		
M1: lnBNF~Temp+Ecosystem	7	348.1	-166.7	0.35	0.55
M2: lnBNF~Temp+Ecosystem+Temp*Ecosystem	8	350.3	-166.6	0.41	0.82
M3: lnBNF~1	5	353.3	-171.4	9.21	0.002
M4: lnBNF~Ecosystem	6	355.2	-171.3	8.98	<0.001

Supplementary references

1 Trimmer, M. *et al.* Riverbed methanotrophy sustained by high carbon conversion efficiency. *The ISME journal* **9**, 2304-2314 (2015).

2 Zappa, C. J. *et al.* Environmental turbulent mixing controls on air-water gas exchange in marine and aquatic systems. *Geophysical Research Letters* **34** (2007).

3 Wanninkhof, R. Relationship between wind speed and gas exchange over the ocean revisited. *Limnology and Oceanography: Methods* **12**, 351-362 (2014).

4 Zhu, Y. *et al.* Disproportionate increase in freshwater methane emissions induced by experimental warming. *Nature Climate Change*, 1-6 (2020).

5 Avnimelech, Y., Ritvo, G., Meijer, L. E. & Kochba, M. Water content, organic carbon and dry bulk density in flooded sediments. *Aquacultural engineering* **25**, 25-33 (2001).

6 Yvon-Durocher, G., Jones, J. I., Trimmer, M., Woodward, G. & Montoya, J. M. Warming alters the metabolic balance of ecosystems. *Philosophical Transactions of the Royal Society of London B: Biological Sciences* **365**, 2117-2126 (2010).

7 Staal, M., Meysman, F. J. & Stal, L. J. Temperature excludes N_2-fixing heterocystous cyanobacteria in the tropical oceans. *Nature* **425**, 504-507 (2003).

8 Lehtimaki, J., Moisander, P., Sivonen, K. & Kononen, K. Growth, nitrogen fixation, and nodularin production by two Baltic Sea cyanobacteria. *Applied and environmental microbiology* **63**, 1647-1656 (1997).

9 Breitbarth, E., Oschlies, A. & LaRoche, J. Physiological constraints on the global distribution of *Trichodesmium* - effect of temperature on diazotrophy. (2007).

10 Falcón, L. I., Pluvinage, S. & Carpenter, E. J. Growth kinetics of marine unicellular N_2-fixing cyanobacterial isolates in continuous culture in relation to phosphorus and temperature. *Marine Ecology Progress Series* **285**, 3-9 (2005).

11 Andersen, K. & Shanmugam, K. Energetics of biological nitrogen fixation: determination of the ratio of formation of H_2 to NH_4^+ catalysed by nitrogenase of *Klebsiella pneumoniae in vivo*. *Microbiology* **103**, 107-122 (1977).

12 Waughman, G. The effect of temperature on nitrogenase activity. *Journal of Experimental Botany* **28**, 949-960 (1977).

13 Ryle, G., Powell, C., Timbrell, M. & Gordon, A. Effect of temperature on nitrogenase activity in white clover. *Journal of Experimental Botany* **40**, 733-739 (1989).

14 Rainbird, R. M., Atkins, C. A. & Pate, J. S. Effect of temperature on nitrogenase functioning in cowpea nodules. *Plant Physiology* **73**, 392-394 (1983).

15 Smith, G. W. & Hayasaka, S. S. Nitrogenase activity associated with Halodule wrightii roots. *Applied and Environmental Microbiology* **43**, 1244-1248 (1982).

16 Rao, V. R. Effect of temperature on the nitrogenase activity of intact and detached nodules in Lotus and Stylosanthes. *Journal of Experimental Botany* **28**, 261-267 (1977).

17 Breheny, P. & Burchett, W. Visualization of regression models using visreg. *R J.* **9**, 56 (2017).

18 McIlvin, M. R. & Altabet, M. A. Chemical conversion of nitrate and nitrite to nitrous oxide for nitrogen and oxygen isotopic analysis in freshwater and seawater. *Analytical Chemistry* **77**, 5589-5595 (2005).

19 Lansdown, K. *et al.* Importance and controls of anaerobic ammonium oxidation influenced by riverbed geology. *Nature Geoscience* **9**, 357-360 (2016).
