# Supplementary material for: Direct biological fixation provides a freshwater sink for N2O
Source: Nat Commun. 2023 Oct 25;14:6775. doi: 10.1038/s41467-023-42481-2 (PMC10600110; doi:10.1038/s41467-023-42481-2)
Supplement: Supplementary file 3 — Reporting Summary [file 41467_2023_42481_MOESM3_ESM.pdf]

nature portfolio

Corresponding author(s): Mark Trimmer

Last updated by author(s): Sep 28, 2023

Reporting Summary

Nature Portfolio wishes to improve the reproducibility of the work that we publish. This form provides structure for consistency and transparency in reporting. For further information on Nature Portfolio policies, see our Editorial Policies and the Editorial Policy Checklist.

Statistics

For all statistical analyses, confirm that the following items are present in the figure legend, table legend, main text, or Methods section.

n/a	Confirmed	
☐	☒	The exact sample size (*n*) for each experimental group/condition, given as a discrete number and unit of measurement
☐	☒	A statement on whether measurements were taken from distinct samples or whether the same sample was measured repeatedly
☐	☒	The statistical test(s) used AND whether they are one- or two-sided<br>*Only common tests should be described solely by name; describe more complex techniques in the Methods section.*
☐	☒	A description of all covariates tested
☒	☐	A description of any assumptions or corrections, such as tests of normality and adjustment for multiple comparisons
☐	☒	A full description of the statistical parameters including central tendency (e.g. means) or other basic estimates (e.g. regression coefficient) AND variation (e.g. standard deviation) or associated estimates of uncertainty (e.g. confidence intervals)
☐	☒	For null hypothesis testing, the test statistic (e.g. *F*, *t*, *r*) with confidence intervals, effect sizes, degrees of freedom and *P* value noted<br>*Give P values as exact values whenever suitable.*
☒	☐	For Bayesian analysis, information on the choice of priors and Markov chain Monte Carlo settings
☒	☐	For hierarchical and complex designs, identification of the appropriate level for tests and full reporting of outcomes
☒	☐	Estimates of effect sizes (e.g. Cohen's *d*, Pearson's *r*), indicating how they were calculated

Our web collection on statistics for biologists contains articles on many of the points above.

Software and code

Policy information about availability of computer code

Data collection	Data for N2O saturation were collected by "GC ChemStation Software rev A.10.02". Data for N2 saturation, total 15N2O reduction, and 15N2 production from 15N2O were collected by "Gas Isotope Ratio MS Software Isodat version 3.0, Thermo Fisher Scientific". Data for the 15N assimilation into the biomass were collected by "Callisto CF-IRMS version 10.0.72, Sercon, UK". Quantitative PCR data was collected using Bio-Rad CFX Manager (version 3.1).
Data analysis	All statistical analyses and plotting were performed in R (version 4.0.3) using RStudio (version 1.3.1093). Generalized additive mixed effects models (GAMMs) were fitted and analysed using the 'lme4' package (version 1.1-26). Quantile regression models were fitted and analysed using the 'quantreg' package (version 5.75). Sequence analysis was conducted using QIIME2 (version 2021.11).

For manuscripts utilizing custom algorithms or software that are central to the research but not yet described in published literature, software must be made available to editors and reviewers. We strongly encourage code deposition in a community repository (e.g. GitHub). See the Nature Portfolio guidelines for submitting code & software for further information.

Data

Policy information about availability of data

All manuscripts must include a data availability statement. This statement should provide the following information, where applicable:

- Accession codes, unique identifiers, or web links for publicly available datasets
- A description of any restrictions on data availability
- For clinical datasets or third party data, please ensure that the statement adheres to our policy

Source data are provided with this paper. The DNA sequences are in the National Center for Biotechnology Information database, under BioProject ID PRJNA984972 [https://www.ncbi.nlm.nih.gov/sra/?term=PRJNA984972].

Research involving human participants, their data, or biological material

Policy information about studies with human participants or human data. See also policy information about sex, gender (identity/presentation), and sexual orientation and race, ethnicity and racism.

Reporting on sex and gender	No human participants were involved in this study.
Reporting on race, ethnicity, or other socially relevant groupings	N/A
Population characteristics	N/A
Recruitment	N/A
Ethics oversight	N/A

Note that full information on the approval of the study protocol must also be provided in the manuscript.

Field-specific reporting

Please select the one below that is the best fit for your research. If you are not sure, read the appropriate sections before making your selection.

☐ Life sciences ☐ Behavioural & social sciences ☒ Ecological, evolutionary & environmental sciences

For a reference copy of the document with all sections, see nature.com/documents/nr-reporting-summary-flat.pdf

Ecological, evolutionary & environmental sciences study design

All studies must disclose on these points even when the disclosure is negative.

Study description	We used our well-replicated (n=20) and well-established (since 2005) outdoor experimental ponds (mesocosms) at Queen Mary's River Laboratory in East Stoke, Dorest, UK. Each pond is approximately 2m in diameter, 0.5m deep and hold ~1000L of water, solely supplied by rainwater. The whole mesocosm facility covers an area of ~130m2 and can be seen on Google Earth at 50° 40' 48.64" N, 2° 11' 09.46" W.<br>We investigated whether these N-limited ponds can act as sinks for the climate-potent gas N2O and whether direct N2O fixation could potentially explain other N2O sinks found in natural waters. With biomass incubations using 15N2O and 15N2 tracers, we distinguished direct from indirect N2O fixation and characterised the temperature responses of N2O and N2 fixation. To characterise N2O and N2 saturation, water samples were collected from all 20 ponds monthly for 11 months from November 2019 to April 2022. To characterise the temperature responses of N2O and N2 fixation, floating and benthic biomass were collected from the ponds monthly for five months from November 2019 to December 2020, with 8 to 10 ponds randomly selected per biomass type and sampling date. 15N2 and 15N2O at 9 µM were added to the 15N2O and 15N2 treatments, while un-amended vials were controls. All vials were incubated at five different temperatures covering a typical temperature range in the pond water (Supplementary Fig. 4). Further, to characterize the potential nifH communities responsible for N2O fixation, floating biomass was collected from 10 ponds in May 2021 and incubated for 25 days amended with 10 µM of N2O or un-amended as controls. We then characterised the nifH gene abundance and communities in relation to total N2O reduction.
Research sample	Our well-replicated (n=20) experimental ponds were designed to mimic shallow freshwater lakes and have previously been reported on for primary production, respiration, methane emissions, and trophic transfer efficiency between 2010 to 2022. The experimental ponds are well-established and provide a good representation of freshwater ecosystems, with various phytoplankton and zooplankton taxa, especially the diverse cyanobacteria communities, making them an ideal environment to explore nitrogen fixation.
Sampling strategy	20 artificial ponds, 10 warmed and 10 at ambient temperature, as above "Study description" and "Spatial Scale" below. As described in the main text we sampled in different months of the year to capture seasonal changes in gas saturation and inorganic nutrient concentrations but due to the covid pandemic travel and accommodation restrictions we could not sample continuously for 12-months starting from late 2019 and had to revisit over multiple years.<br>Samples for N2O and N2 saturation were collected from all 20 ponds for 11 months to characterise whether the ponds typically act as sinks for N2O and N2, and to characterise the seasonality of the two N gases. The samples for the biomass incubations were

	collected from randomly selected 8 to 10 ponds for two biomass types (floating and benthic), three treatments (15N2O, 15N2, Control), five temperatures, and five months to guarantee representative overall estimates. Samples for 25-day incubation for the nifH community analysis were collected from randomly selected 10 ponds. We did not do any a priori statistical power analysis or similar to determine sample size as the experimental ponds are fixed at 20 replicates. That said, we have published numerous papers already that clearly show they provide a good level of experimental replication.
Data collection	Data for N2O saturation (Fig. 1a, 1b, 1d) were collected by a gas chromatograph fitted with a µECD (Agilent Technology UK Ltd., South Queensferry, UK) and recorded by the chromatography data system "GC ChemStation Software rev A.10.02". Data for total inorganic nitrogen and soluble reactive phosphorous (Fig. 1f,1g and Fig. 3b) were collected by an automated wet chemistry analyzer (San++, SKALAR Analytical B.V.) and recorded by software “FlowAccess”. The temperature data for pond water (Fig. 1d and 1e) were collected by HQD portable meter (Hach) in situ and recorded by pen and paper. The temperature data for incubation (Fig. 4a) were collected by a data logger (HOBO Pendant, Onset) and recorded by software “HOBOware”. Data for N2 saturation (Fig. 1a, c, e), total 15N2O reduction (Fig. 3a), and 15N2 production from 15N2O (Fig. 2c and Fig. 4b) were collected by a continuous flow isotope ratio mass spectrometer (CF-IRMS, Delta V Plus, Thermo Finnigan) and recorded by the software "Gas Isotope Ratio MS Software Isodat version 3.0, Thermo Fisher Scientific". Data for the 15N assimilation into the biomass (Fig. 2a and 2b) were collected by an isotope ratio mass spectrometry (IRMS) (Sercon Integra 2 instrument) and recorded by "Callisto CF-IRMS version 10.0.72, Sercon, UK". The data described above were collected by Yueyue Si.<br>Data for N2O reduction (Fig. 5a) were collected and recorded as per above. Quantitative PCR data (presented in result section “nifH communities in relation to N2O reduction”) was collected by CFX96 Touch Real-Time PCR cycler and recorded by Bio-Rad CFX Manager (version 3.1). The data described above were collected by Yizhu Zhu.<br>The sequencing data was collected by Illumina MiSeq platform (300 base-pair paired-end) by Barts and The London Genome Centre at Queen Mary University of London.
Timing and spatial scale	As above, the experimental ponds (mesocosms) were established at Queen Mary University of London River Laboratory in 2005. Each pond is approximately 2m in diameter, 0.5m deep and hold ~1000L of water, solely supplied by rainwater. The whole mesocosm facility covers an area of ~130m2 and can be seen on Google Earth at 50° 40' 48.64" N, 2° 11' 09.46" W.<br>Samples for N2O and N2 saturation were collected from all 20 ponds at 12pm to 3pm monthly for 11 months from November 2019 to April 2022. Samples for the biomass incubations with 15N2O and 15N2 were collected monthly for five different months from November 2019 to December 2020, and all samples were incubated for 24 hours. Samples for characterising the nitrogenase, nifH communities were collected in May 2021, and incubated for 0, 3, 10, and 25 days.
Data exclusions	Data for the rate of total 15N2O reduction and 15N assimilation were strongly skewed, potentially due to normalizing the rate to a unit of dry biomass which may not account for the abundance of N2 and N2O fixing microbes. Therefore, the outliers beyond the 95th percentile were removed from the presentation of total 15N2O reduction and 15N assimilation. Data for the annual daily average temperature in the ponds were excluded from two out of 20 ponds due to the failure of the temperature logger. The nutrient and gas saturation data collected for February 2020, were omitted from further analysis, due to the exceptionally heavy rainfall before the sampling date.
Reproducibility	The experimental ponds are well replicated (n=20). Samples for N2O and N2 saturation were collected monthly from all 20 ponds for 11 months from November 2019 to April 2022 to guarantee reproducibility. The floating and benthic biomass in the 15N2O and 15N2 incubations were collected monthly from 8 to 10 ponds, randomly selected, for five months from November 2019 to December 2020, to improve the overall estimates of total N2O reduction and 15N2O and 15N2 assimilation. For the nitrogenase, nifH community analyses, the biomass was collected from 10 randomly selected ponds to give a representative result.
Randomization	The ponds were randomly selected for sampling biomass for incubations. The incubation vials were randomly ordered for the addition of 15N2O and 15N2 tracers. Samples for the analysis of total N2O reduction, 15N2 production from 15N2O, and assimilation of 15N2O and 15N2 into the biomass were analysed in random orders.
Blinding	The data and analyses were largely performed by Yueyue Si as part of their PhD thesis including: samples for N2O and N2 saturation, nutrients and 15N-tracer incubations, and, as such, blinding is not appropriate. Similarly, Yizhu Zhu collected and processed the samples for the 25-day incubation used for the nifH community analysis as part of her Postdoc, hence, again, blinding was not possible or appropriate.

Did the study involve field work? ☐ Yes ☒ No

Reporting for specific materials, systems and methods

We require information from authors about some types of materials, experimental systems and methods used in many studies. Here, indicate whether each material, system or method listed is relevant to your study. If you are not sure if a list item applies to your research, read the appropriate section before selecting a response.

Materials & experimental systems

n/a	Involved in the study
☒	☐ Antibodies
☒	☐ Eukaryotic cell lines
☒	☐ Palaeontology and archaeology
☒	☐ Animals and other organisms
☒	☐ Clinical data
☒	☐ Dual use research of concern
☒	☐ Plants

Methods

n/a	Involved in the study
☒	☐ ChIP-seq
☒	☐ Flow cytometry
☒	☐ MRI-based neuroimaging
